# Treatment strategies for new onset atrial fibrillation in patients treated on an intensive care unit: a systematic scoping review

**DOI:** 10.1186/s13054-021-03684-5

**Published:** 2021-07-21

**Authors:** Laura Drikite, Jonathan P. Bedford, Liam O’Bryan, Tatjana Petrinic, Kim Rajappan, James Doidge, David A. Harrison, Kathryn M. Rowan, Paul R. Mouncey, Duncan Young, Peter J. Watkinson, Mark Corbett

**Affiliations:** 1 Nuffield Department of Clinical Neurosciences, University of Oxford, Oxford, UK; 2 Intensive Care National Audit and Research Centre (ICNARC), 24 High Holborn, London, WC1V 6AZ UK; 3 NIHR Biomedical Research Centre, Nuffield Department of Clinical Neurosciences, University of Oxford, Oxford University Hospitals NHS Trust, Oxford, UK; 4 Cairns Library, University of Oxford Health Care Libraries, Oxford, UK; 5 Centre for Reviews and Dissemination, University of York, York, UK; 6 Cardiac Department, John Radcliffe Hospital, Oxford University Hospitals NHS Foundation Trust, Oxford, UK

**Keywords:** New onset atrial fibrillation, Intensive care, Critical care, Scoping review, Arrhythmia, Stroke

## Abstract

**Background:**

New-onset atrial fibrillation (NOAF) in patients treated on an intensive care unit (ICU) is common and associated with significant morbidity and mortality. We undertook a systematic scoping review to summarise comparative evidence to inform NOAF management for patients admitted to ICU.

**Methods:**

We searched MEDLINE, EMBASE, CINAHL, Web of Science, OpenGrey, Cochrane Database of Systematic Reviews, Cochrane Central Register of Controlled Trials, Database of Abstracts of Reviews of Effects, ISRCTN, ClinicalTrials.gov, EU Clinical Trials register, additional WHO ICTRP trial databases, and NIHR Clinical Trials Gateway in March 2019. We included studies evaluating treatment or prevention strategies for NOAF or acute anticoagulation in general medical, surgical or mixed adult ICUs. We extracted study details, population characteristics, intervention and comparator(s), methods addressing confounding, results, and recommendations for future research onto study-specific forms.

**Results:**

Of 3,651 citations, 42 articles were eligible: 25 primary studies, 12 review articles and 5 surveys/opinion papers. Definitions of NOAF varied between NOAF lasting 30 s to NOAF lasting > 24 h. Only one comparative study investigated effects of anticoagulation. Evidence from small RCTs suggests calcium channel blockers (CCBs) result in slower rhythm control than beta blockers (1 study), and more cardiovascular instability than amiodarone (1 study). Evidence from 4 non-randomised studies suggests beta blocker and amiodarone therapy may be equivalent in respect to rhythm control. Beta blockers may be associated with improved survival compared to amiodarone, CCBs, and digoxin, though supporting evidence is subject to confounding. Currently, the limited evidence does not support therapeutic anticoagulation during ICU admission.

**Conclusions:**

From the limited evidence available beta blockers or amiodarone may be superior to CCBs as first line therapy in undifferentiated patients in ICU. The little evidence available does not support therapeutic anticoagulation for NOAF whilst patients are critically ill. Consensus definitions for NOAF, rate and rhythm control are needed.

**Supplementary Information:**

The online version contains supplementary material available at 10.1186/s13054-021-03684-5.

## Background

New onset atrial fibrillation (NOAF), usually defined as atrial fibrillation (AF) occurring in patients with no known history of AF [1], is a common arrhythmia in critically ill patients [2]. NOAF occurs in 5–11% of patients admitted to an intensive care unit (ICU) [3–6], and up to 46% of patients with septic shock [7, 8]. NOAF in critically ill patients can cause cardiovascular instability [5] and is associated with increased risk of thromboembolism [9], increased mortality [10] and length of ICU stay [11], and higher healthcare costs [11].

Guidelines for management of AF [12, 13] do not directly apply to critically ill patients. NOAF in patients treated on an ICU differs from AF in patients in the community in terms of causes of rhythm disturbance [14, 15], risks and effectiveness of treatments [16]. The lack of evidence for managing NOAF in patients treated on an ICU means treatment practice differs widely [17].

We conducted a scoping review to provide an overview of current evidence for the effectiveness and safety of pharmacological, electrical, and other non-pharmacological NOAF treatments, prophylactic strategies, and acute anticoagulation for stroke prophylaxis in critically ill patients. We also aimed to describe commonly used definitions of NOAF in patients treated on an ICU and suggest recommendations and barriers for future research.

A recent scoping review described the incidence, risk factors, outcomes and management strategies related to NOAF during critical illness [10]. It included patients with pre-existing AF and studies conducted outside ICUs. Our review focusses on the comparative evidence for treatment of NOAF in patients treated on an ICU.

## Materials and methods

### Search and identification of studies

We developed our search strategy with an information specialist (TP) in MEDLINE with no date or language restrictions. We included terms used for NOAF combined with terms used for intensive care (see Additional file 1).

We adapted the MEDLINE search strategy to identify papers in the following databases in March 2019: MEDLINE, EMBASE, CINAHL, Web of Science (including Conference Proceedings Citation Index: Science), OpenGrey, the Cochrane Database of Systematic Reviews, the Cochrane Central Register of Controlled Trials (CENTRAL), and the Database of Abstracts of Reviews of Effects (DARE) to 2015. The following clinical trial databases were searched for studies in progress, or completed but not reported: ISRCTN, ClinicalTrials.gov, the EU Clinical Trials register, additional WHO ICTRP trial databases, and the NIHR Clinical Trials Gateway.

### Eligibility criteria

We included studies of adults (age ≥ 16 years) in general medical, surgical or mixed ICUs. We excluded studies of cohorts defined by a single disease or narrow disease group not normally admitted to a general ICU, and studies based on service-specific ICUs. We included studies of pharmacological, electrical and other non-pharmacological treatment strategies for treatment or prophylaxis of NOAF and the use of acute anticoagulation. The outcomes of interest were rhythm and rate control, length of ICU and hospital stay, mortality (ICU, hospital, 30-day, long term), arterial thromboembolism and adverse treatment effects. Quantitative studies, reviews, practitioner surveys, and opinion pieces were eligible for this review.

### Study selection and data charting

We used EPPI-Reviewer 4 software (Evidence for Policy and Practice Information and Co-ordinating Centre, University of London, London, UK) to identify duplicate records and for title and abstract screening. Two reviewers (LD and LOB) independently screened titles, abstracts and full-text articles, with discrepancies resolved through discussion or by a third reviewer (MC).

We also reviewed reference lists of included studies for further relevant citations. Full-text articles not published in English were screened by native speakers.

We developed data charting forms (see Additional file 1: Tables S1–S10) for the following study designs: randomised controlled trials (RCTs), prospective comparative studies, retrospective comparative studies, and non-comparative studies. The extracted data included: details of the study, population characteristics, description of intervention and comparator(s), methods to address confounding, results, and recommendations for future research.

Decisions about which population characteristics to extract were informed by a systematic review on risk factors for NOAF on the ICU [18] and a retrospective observational study on predictors for sustained NOAF in the critically ill [19]. Data were extracted by one reviewer (LD) and checked by another (JB); disagreements were referred to a third reviewer (MC).

### Critical appraisal

We evaluated RCTs using version 2 of the Cochrane risk of bias tool [20]. We evaluated non-randomised comparative studies for risk of bias using the ROBINS-I tool [21] if they were reported as full papers, included at least 100 patients per treatment arm and reported on methods to adjust for confounding.

Studies which did not meet these criteria were deemed to be at a critical risk of bias. The ROBINS-I tool was adapted by including a stopping rule: the assessment stopped if a serious, or critical, risk of bias judgement was made for the ‘bias due to confounding’ domain. For the confounding domain, decisions regarding which covariates should be reported as being controlled for in analyses were made by the clinical experts in the CAFE study team and are reported in Additional file 1 along with the risk of bias judgements (see Additional file 1: Tables S11).

### Collating and summarising results

We presented details of the primary studies in structured tables categorised by pairwise drug comparison and by study design. For each type of study design, we described the extent, range and nature of the identified research. Study parameters and results were then described and summarised narratively.

## Results

### Search results

**Fig. 1:**
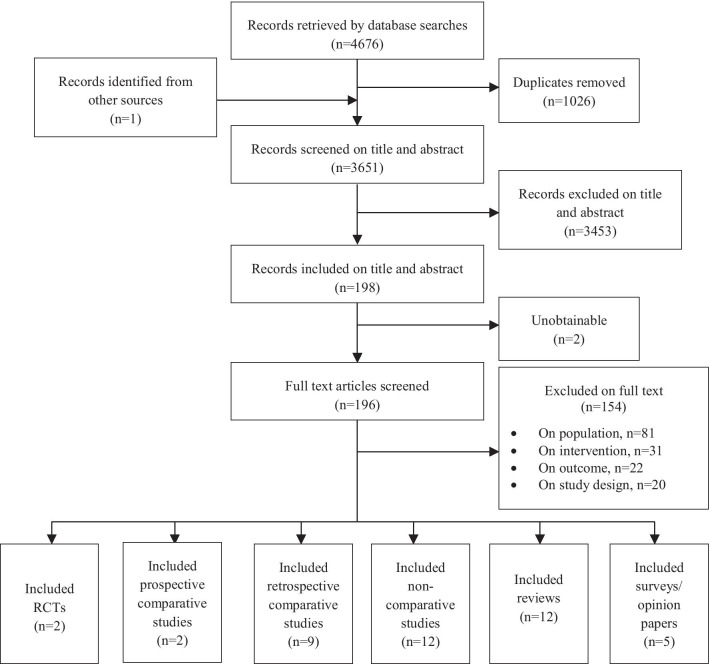
Flow chart showing the number of studies identified, excluded and eligible for inclusion in the scoping review

Of the 3651 articles screened on title and abstract, 198 articles were identified as being of potential interest and screened on full text. After full text screening, 42 articles were included in the review: 25 primary studies, 12 review articles and 5 surveys/opinion papers. Of the 25 primary studies, two RCTs [22, 23] two prospective comparative studies [24, 25], nine retrospective comparative studies [26–34] and 12 non-comparative studies [5, 35–45] were included. Six studies [27–30, 32, 39] were available only as conference abstracts. Figure 1 illustrates the flow of the articles through the review process.

### Characteristics of included studies

Nine studies [22, 24, 27, 31, 35, 37, 41, 43, 44] were conducted in speciality ICUs such as surgical, trauma, or medical. Five studies [5, 23, 38, 42, 45] were conducted in mixed ICUs and one study [26] in a general ICU. The type of ICU was not specified in 10 studies [25, 28–30, 32–34, 36, 39, 40]. Eleven studies included patients with sepsis [5, 27, 28, 33–35, 38, 43] or septic shock [25, 26, 29] as primary diagnoses. Four studies [22, 24, 31, 41] were conducted in a noncardiac surgical population. Two studies included noncardiac and cardiac surgery patients [37, 44] and one study was conducted in surgical population; however, the type of surgery was not specified [40].

Nineteen studies [5, 22–24, 26–28, 30–33, 35, 36, 38, 40, 41, 43–45] investigated the treatment effects of pharmacological treatments, two studies [25, 29] looked at prophylactic treatments, and two studies [37, 42] investigated electrical treatments. One study [39] reported on both pharmacological treatments and anticoagulation for stroke prophylaxis. One study [34] on anticoagulation was included in the review.

Overview of the primary study evidence by intervention and study design can be found in Additional file 1: Tables S12.

### Definitions used for NOAF

Studies varied in how they reported and defined NOAF. Five studies [5, 31, 35, 37, 44] defined NOAF as having AF with a heart rate of > 100 beats per minute and two studies [23, 32] used a heart rate threshold of > 120 beats per minute. Seven studies [22, 23, 25, 35, 37, 43, 44] reported different time periods for which NOAF must be sustained, ranging from 30 s to 24 h. Six studies [5, 31, 33, 34, 40, 42] specified in which instances AF would be considered as ‘new onset’. For example, when a patient had no prior history of AF [31], without previous history of atrial tachyarrhythmias and antiarrhythmic drug use [40], and AF not present on admission [33, 34]. Ten studies [24, 26–30, 36, 38, 39, 41] did not provide any definition for NOAF.

### Results from comparative studies

#### Amiodarone versus beta blockers

**Fig. 2:**
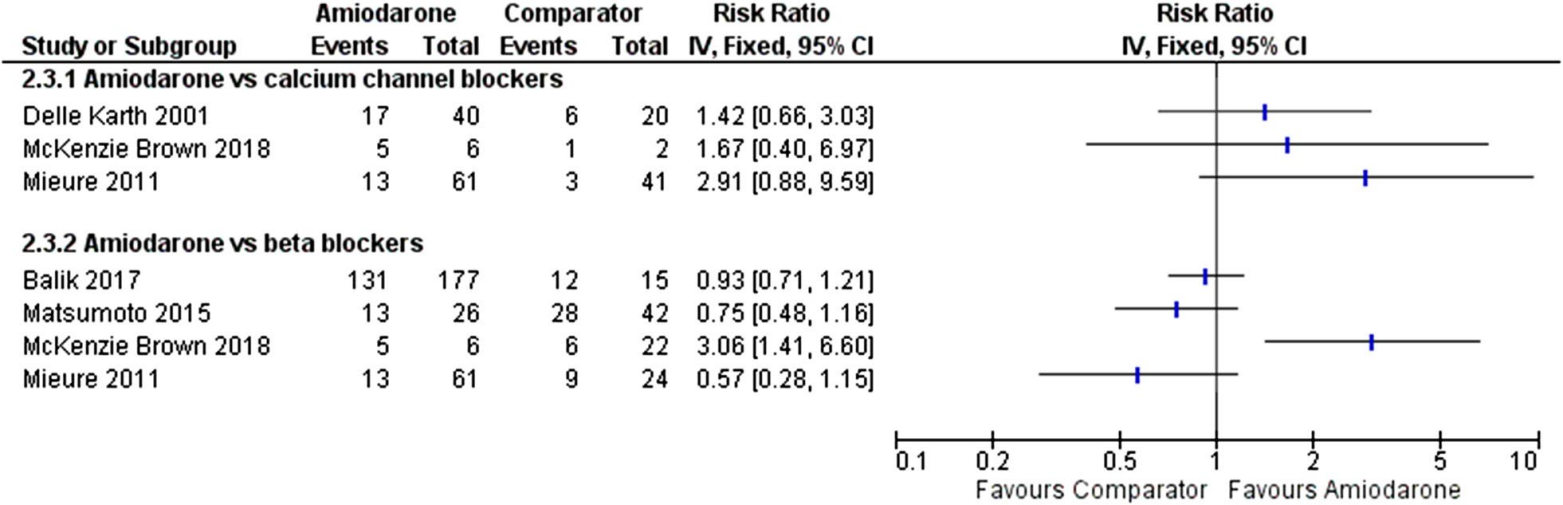
Rhythm control risk ratio results for studies comparing amiodarone with beta blockers or calcium channel blockers

Six retrospective comparative studies [26, 28, 30–33] compared amiodarone with beta blockers (Table 1, Additional file 1: Tables S5 and S6). Most studies did not report on doses [28, 31–33] or modes of administration [28, 31, 32]. A large study [33] of 39,693 patients with sepsis reported that patients treated with amiodarone were more likely to be critically ill with septic shock than patients treated with beta blockers. After adjustment for confounding, beta blockers were associated with lower mortality than amiodarone (RR 0.67, 95% CI: 0.59–0.77) [33]. However, only 60% of patients were on an ICU. Therefore, this study’s results may not be applicable to a broad ICU population. The study was also judged as being at a serious risk of bias due to confounding (Additional file 1). Balik et al. [26] showed higher but not statistically significant ICU mortality in patients receiving amiodarone (40%) than in patients receiving metoprolol (21%) [26]. A conference abstract by Jaffer et al. [28] also reported no statistically significant difference in mortality. Four studies [26, 30–32] compared conversion rates between amiodarone and beta-blockers. Three studies [26, 30, 32] showed no statistically significant difference in cardioversion rates between the treatments. Balik et al. [26] did not adjust for confounding factors such as illness severity [26]. Two studies did not report on the methods used for the analysis [30, 32]. No meaningful conclusions from the results of Brown et al. could be made with only 6 patients receiving amiodarone [31]. Figure 2 shows rhythm control risk ratio results for studies comparing amiodarone with beta blockers. Although Fig. 2 enables a crude comparison of results, the studies were too heterogeneous for this to depict a true comparison. It should also be noted that only studies which reported numerators and denominators for the rhythm control outcome could be included in Fig. 2.

**Table 1:** Studies comparing amiodarone and beta blockers

Authors	Sample size and setting	Primary diagnosis	Study design and risk of bias	Intervention	Rate control outcome	Rhythm control outcome	Mortality outcome
Walkey et al. (2016)	*n* = 3174^a^ (NOAF patients) Setting: USA	Sepsis	Retrospective comparative Risk of bias: Serious	Beta blockers (metoprolol, esmolol, atenolol, labetalol, propranolol) versus amiodarone	Not assessed	Not assessed	Hospital: RR^b^ 0.67 (95% CI 0.59–0.77)
Matsumoto et al. (2015) (conference abstract)	*n* = 276 *n* = 116 (amiodarone) *n* = 160 (landiolol) Setting: Japan ICU	Not reported	Retrospective comparative Risk of bias: Critical	Amiodarone versus landiolol	Not assessed	NS^c^ Amiodarone: 50% Landiolol: 67%	Not assessed
Balik et al. (2017)	*n* = 234^d^ *n* = 177 (amiodarone) *n* = 15 (metoprolol) Setting: Czech Republic general ICU	Septic shock	Retrospective comparative Risk of bias: Critical	Amiodarone versus metoprolol	Not assessed	Amiodarone: 74% Metoprolol: 92%	ICU: NS Hospital: NS
Mieure et al. (2011) (conference abstract)	*n* = 126^e^ *n* = 61 (amiodarone) *n* = 24 (metoprolol) Setting: USA ICU	Not reported	Retrospective comparative Risk of bias: Critical	Amiodarone versus metoprolol	< 100 bpm within 24 h from initiation of treatment: *p* = 1.00 Amiodarone: 85.2% Metoprolol: 87.5%	*p* = 0.013 Amiodarone: 21.3% Metoprolol: 37.5%	Not assessed
Jaffer et al. (2016) (conference abstract)	*n* = 65 Setting: USA ICU	Septic shock	Retrospective comparative Risk of bias: Critical	Amiodarone versus beta blockers (drug not specified)	Not assessed	Not assessed	NS
McKenzie Brown et al. (2018)	*n* = 33^e^ *n* = 6 (amiodarone) *n* = 22 (beta blockers) Setting: USA surgical ICU	Noncardiac surgical population	Retrospective comparative Risk of bias: critical	Amiodarone versus beta blockers (drug not specified)	*p* = 0.001 Amiodarone: 83% Beta blockers: 27%^f^	*p* = 0.001 Amiodarone: 83% Beta blockers: 27%^f^	Not compared between treatment groups

^a^Includes calcium channel blockers and digoxin groups

^b^Relative risk

^c^Statistically not significant

^d^Includes propafenone group

^e^Includes calcium channel blockers and no treatment groups

#### Amiodarone versus calcium channel blockers

One RCT [23], one prospective [24] and three retrospective comparative studies [28, 31, 32] compared amiodarone and calcium channel blockers (Table 2, Additional file 1: Tables S1, S2, S3, S4, S5, and S6), all were relatively small with between eight and 61 patients included.

**Table 2:** Studies comparing amiodarone and calcium channel blockers

Authors	Sample size and setting	Primary diagnosis	Study design and risk of bias	Intervention	Rate control outcome	Rhythm control outcome	Mortality outcome
Delle Karth et al. (2001)	*n* = 60 *n* = 20 (diltiazem) *n* = 20 (amiodarone bolus) *n* = 20 (amiodarone bolus and 24 h infusion) Setting: Austria ICU	Mixed cardiac and medical ICU population	RCT^a^ Risk of bias: high	Diltiazem versus amiodarone bolus versus amiodarone bolus and 24 h continuous infusion	Rate reduction within 4 h: NS^b^ Diltiazem: 70% Amiodarone bolus: 55% Amiodarone bolus with 24 h continuous infusion: 75% Rate reduction within 24 h: Diltiazem versus amiodarone groups: * p* = .001 Amiodarone bolus versus amiodarone bolus with 24 h continuous infusion: * p* = .08	Within 4 h: NS Diltiazem: 30% Amiodarone bolus: 40% Amiodarone bolus with 24 h continuous infusion: 45%	Not assessed
Gerlach et al. (2008)	*n* = 61 *n* = 55 NOAF patients *n* = 28 (diltiazem) *n* = 27 (amiodarone) Setting: USA Surgical ICU	Noncardiac surgical population	Prospective comparative Risk of bias: Critical	Diltiazem versus amiodarone	Not assessed	At 24 h: NS Diltiazem: 87% Amiodarone: 87% Mean time to conversion: NS Diltiazem: 7 h Amiodarone: 5 h	Not assessed
Jaffer et al. (2016) (conference abstract)	*n* = 65 Setting: USA ICU	Septic shock	Retrospective comparative Risk of bias: Critical	Calcium channel blockers (drug not specified) versus amiodarone	Not assessed	Not assessed	NS
Mieure et al. (2011) (conference abstract)	*n* = 126^c^ *n* = 61 (amiodarone) *n* = 41 (diltiazem) Setting: USA ICU	Not reported	Retrospective comparative Risk of bias: Critical	Diltiazem versus amiodarone	At 24 h: NS Diltiazem: 85% Amiodarone: 85%	Diltiazem: 7% Amiodarone: 21%	Not assessed
McKenzie Brown et al. (2018)	*n* = 33^d^ *n* = 6 (amiodarone) *n* = 2 (calcium channel blockers) Setting: USA Surgical ICU	Noncardiac surgical population	Retrospective comparative Risk of bias: Critical	Calcium channel blockers (drug not specified) versus amiodarone	Amiodarone: 83% Calcium channel blockers: 50%	Amiodarone: 83% Calcium channel blockers: 50%	Not assessed

^a^Randomised controlled trial

^b^Statistically not significant

^c^Includes beta blockers group

^d^Includes beta blockers group no treatment groups

A small RCT of 60 patients compared diltiazem, amiodarone bolus and amiodarone bolus followed by an amiodarone infusion in a mixed ICU population [23]. No evidence of a difference was identified between treatment groups in the primary study endpoint of rate reduction ≥ 30% at 4 h. Hypotension resulting in drug discontinuation was more common with diltiazem use (30%) vs amiodarone (0–5%). This study [23] was judged to have a high risk of bias. A non-randomised study comparing diltiazem and amiodarone in a noncardiac surgical ICU population found no evidence of differences between the study groups in the proportion cardioverted at 24 h or in time to cardioversion [24]. No evidence of a difference in rates of hypotension was identified. Similar length of ICU and hospital stays was also reported. This study [24] was small and most likely underpowered to detect any treatment differences. A conference abstract found no statistically significant difference in mortality at discharge [28]. Two studies [31, 32] compared rate and rhythm control between treatment groups but results were subject to much uncertainty due to the limited data reported [32] and small sample size [31]. Figure 2 shows rhythm control risk ratio results for studies comparing amiodarone with calcium channel blockers.

#### Beta blockers versus calcium channel blockers

One RCT [22] and three retrospective comparative studies [28, 31, 33] compared beta blockers with calcium channel blockers (Table 3, Additional file 1: Tables S1, S2, S5 and S6). Balser et.al [22] conducted a RCT (*n* = 55) comparing esmolol with diltiazem in a noncardiac surgical population. Conversion to sinus rhythm was more common in the esmolol group at 2 h (59% vs 33%, *p* = 0.049); however there was no statistically significant difference at 12 h. There was also no evidence of a difference in hospital mortality [22]. This RCT was judged as having some concerns about possible bias primarily due to the lack of reporting of randomisation methods and the lack of blinding (Additional file 1). Another study [31] compared conversion rates in a surgical ICU population but the sample size was too small to make any conclusions. Two retrospective comparative studies [28, 33] reported no evidence of a difference in hospital mortality.

**Table 3:** Studies comparing beta blockers and calcium channel blockers

Authors	Sample size and setting	Primary diagnosis	Study design and risk of bias	Intervention	Rate control outcome	Rhythm control outcome	Mortality outcome
Balser et al. (1998)	*n* = 55 *n* = 28 (esmolol) *n* = 27 (diltiazem) Setting: USA ICU	Noncardiac surgical population	RCT^a^ Risk of bias: Some concerns	Esmolol versus diltiazem	Not assessed	Within 2 h: NS^b^ Esmolol: 59% Diltiazem: 27%	Hospital: NS Esmolol: 31% Diltiazem: 38%
Walkey et al. (2016)	*n* = 3,174^c^ (NOAF patients) Setting: USA	Sepsis	Retrospective comparative Risk of bias: Serious	Beta blockers (metoprolol, esmolol, atenolol, labetalol, propranolol) versus calcium channel blockers (diltiazem, verapamil)	Not assessed	Not assessed	Hospital: RR^d^ 0.99 (95% CI: 0.86–1.15)
Jaffer et al. (2016) (conference abstract)	*n* = 65 Setting: USA ICU	Septic shock	Retrospective comparative Risk of bias: Critical	Beta blockers versus calcium channel blockers (drugs not specified)	Not assessed	Not assessed	NS
McKenzie Brown et al. (2018)	*n* = 33^e^ *n* = 22 (beta blockers) *n* = 2 (calcium channel blockers) Setting: USA Surgical ICU	Noncardiac surgical population	Retrospective comparative Risk of bias: critical	Beta blockers versus calcium channel blockers (drugs not specified)	Beta blockers: 27% Calcium channel blockers: 50%	Beta blockers: 27% Calcium channel blockers: 50%	Not assessed

Randomised controlled trial

Statistically not significant

Includes amiodarone and digoxin groups

Relative risk

Includes amiodarone group and no treatment groups

#### Beta blockers versus digoxin

One large retrospective study [33] investigated the outcomes in patients who received digoxin versus patients who received beta blockers (Additional file 1: Tables S5, S6, S13, and S4). Following propensity score matching (*n* = 1932), hospital mortality was lower in patients who received beta-blockers compared to patients who received digoxin (RR 0.75 (95% CI: 0.64–0.88)). The study was judged as being at a serious risk of bias due to confounding (Additional file 1).

#### Hydrocortisone versus no treatment

One prospective study [25] and one retrospective study [29] compared hydrocortisone as a prophylactic treatment with no treatment (Table 4, Additional file 1: Tables S3, S4, S5 and S6). Both studies [25, 29] were conducted in patients with septic shock. Launey et al. (2019) reported that the unadjusted ICU and 28-day mortality in the hydrocortisone group was higher than when compared to the no treatment group (37% versus 24% (*p* = 0.018); and 38% versus 26% (*p* = 0.036), respectively), noting that patients who received hydrocortisone were more severely ill than those who did not receive hydrocortisone [25]. However, in the propensity score-weighted analysis, patients who received hydrocortisone were less likely to develop NOAF compared to patients who did not [risk difference 11.9%, RR 0.58 (95% CI 0.35–0.98)] [25]. This study [25] was judged to have serious risk of bias due to missing covariates in the propensity score matching. Similarly, the retrospective study [29] concluded that administering hydrocortisone was associated with a reduction in NOAF incidence. No evidence of a difference in mortality between the study groups was reported [29]. However, this study [29] was published as a conference abstract with limited data available and the dose of hydrocortisone was not reported [29].

**Table 4:** Studies comparing hydrocortisone and no treatment

Authors	Sample size and setting	Primary diagnosis	Study design and risk of bias	Intervention	Incidence of NOAF	Mortality outcome
Launey et al. (2019)	*n* = 261 *n* = 123 (hydrocortisone) *n* = 138 (no treatment) Setting: France ICU	Septic shock	Prospective comparative Risk of bias: serious	Hydrocortisone vs no treatment	RD^a^ − 11.9% (95% CI − 23.4% to − 0.5%) RR^b^ 0.58 (95% CI 0.35–0.98)	ICU: NS^c^ Hydrocortisone: 37% No treatment: 24% 28-day: NS Hydrocortisone: 38% No treatment: 26%
Kane and Hanes (2014) (conference abstract)	*n* = 109 *n* = 39 (hydrocortisone) Setting: USA ICU	Septic shock	Retrospective comparative Risk of bias: critical	Hydrocortisone vs no treatment	*p* = 0.006 Hydrocortisone: 20.5% No treatment: 42.9%	NS

Risk difference

Relative risk

Statistically not significant

#### Anticoagulation versus no treatment

Published comparative evidence for anticoagulation was very limited. One large retrospective study [34] found no benefit from in-hospital anticoagulation for NOAF in sepsis but was at high risk of bias (Additional file 1: Tables S5, S6 and S14). The study (*n* = 38,582 with any AF, *n* = 7522 with NOAF) [34] included hospitalised patients, around 60% of whom were treated on an ICU. Rates of in-hospital stroke were low in the NOAF cohort (1.9%). As the hospital length of stay was not reported, the duration of exposure was unclear [34]. Following propensity score matching (*n* = 5585 analysed), rates of in-hospital ischaemic stroke events and risk of bleeding did not differ significantly between patients who did, and did not, receive parenteral anticoagulation. Given the low event rate, the study [34] may have had inadequate power to determine whether a significant difference exists.

#### Results from non-comparative studies

The following pharmacological treatments were investigated in non-comparative studies: amiodarone [5, 39, 41, 43–45], magnesium-amiodarone step-up scheme [40], beta-blockers [5, 38, 41, 43], calcium channel blockers [5, 41, 43], digoxin [5, 41, 43], and ibutilide [35, 36]. Two non-comparative studies looked at electrical treatments [37, 42].

Two studies investigated anticoagulant therapy. One study reported a 5% risk of major bleeding with IV heparin, though thromboembolism events were not reported [39]. Another study reported a 9% (5/58) risk of major bleeding with therapeutic anticoagulation in patients with NOAF and pre-existing AF [5] with no strokes occurring during ICU admission.

Details of non-comparative studies are reported in Additional file 1: Tables S7, S8, S9 and S10.

#### Results from review articles, surveys, and opinion pieces

A full summary of the reviews, surveys and opinion pieces identified in the review is reported in Additional file 1.

#### Reported recommendations for future research

Most studies and review articles that were included in this review concluded that further prospective research accounting for confounding factors is required to determine the success and clinical implications of prophylactic and treatment strategies in patients treated in an ICU with NOAF [5, 6, 14, 22, 24, 25, 28, 29, 32, 33, 38, 40, 41, 43, 46–52]. It was emphasised that optimal regimens and best dosing strategies for treatments are yet to be established [31, 37, 45]. Eight studies [23, 26, 27, 30, 35, 36, 39, 44] and four review articles [53–56] did not provide any recommendations for future research.

## Discussion

The evidence base for NOAF management for patients in ICU was limited. Many studies identified in this scoping review were non-comparative studies (i.e. lacking a comparator group, *n* = 12). Of the 25 primary studies included in the review only two were RCTs [22, 23] and only three of the non-randomised comparative studies [25, 33, 34] attempted to control for confounding factors. In the studies which used more robust approaches, there were nevertheless still concerns about how bias (arising from their designs and/or analyses) might affect their results. Moreover, considerable heterogeneity defining NOAF, treatment doses (e.g. total dose ranging from less than a gram to eight grams for amiodarone) [5, 23, 24, 26, 30, 44, 45], administration (e.g. bolus or continuous infusion), and timepoints to assess conversion to sinus rhythm (e.g. within two [22], four [23], 12 [23], and 24 h [5, 24, 26, 32] was observed across studies. Similarly, a systematic review was not able to make evidence-based recommendations for pharmacologic rhythm conversion strategies for patients who develop NOAF in a general ICU due to considerable methodological heterogeneity of the included studies [48]. There is therefore a need to establish optimal treatment dosing and administration regimens, as well as standardised and validated outcome measures of treatment success.

The limited evidence from this review [26, 30, 32] suggests that beta-blockers may be equivalent to amiodarone for rhythm control. Where reduced mortality in those who received beta-blockers compared to those who received amiodarone was reported [26, 33], there were significant concerns about bias. Despite this, some review articles [46, 50, 51] argued that beta blockers may be a reasonable first-choice treatment due to the current evidence of decreased mortality [46], and improved heart rate control [46, 50]. Two opinion pieces [14, 57] also favoured the use of beta-blockers as the initial pharmacotherapy, given the limited and indirect evidence. In contrast, five reviews discussed amiodarone as a potentially effective treatment [47, 49, 52–54], though it was also recognised that amiodarone has potentially significant side effects [47, 52, 54].

Calcium channel blockers appeared to be less effective for conversion to sinus rhythm when compared with beta blockers, and result in more hypotension than amiodarone [22, 23]. Two studies [25, 29] reported that hydrocortisone may be effective as a prophylactic treatment. However, these results are subject to much uncertainty due to methodological limitations.

International guidelines [12, 13] provide advice regarding the management of patients presenting acutely with AF, and/or patients with AF with haemodynamic instability. However, the evidence base and expert consensus on which these guidelines are based does not appear to include patients in the intensive care unit setting. Therefore, whilst they may be used to guide some general aspects of AF management in any patient, such as the recommendation to use cardioversion if the patient is acutely haemodynamically unstable, recommendations regarding pharmacological therapy and whether or not the patient should be anticoagulated, either short or long-term, may not apply to this specific patient population.

Comparative evidence for or against electrical cardioversion for patients in ICU with NOAF was not identified in our review. Electrical cardioversion should be considered in patients where AF is contributing to marked haemodynamic instability. NOAF often occurs alongside haemodynamic instability but is more likely to be a significant contributor where ventricular rates are very high or where there is underlying structural heart disease. As with other treatments, electrical cardioversion should be used alongside aggressive management of underlying AF drivers. Further procedural considerations are detailed elsewhere [58].

It is unclear whether to administer therapeutic anticoagulation in critically ill patients with NOAF for stroke prevention. Limited evidence suggests bleeding risk outweighs the increased risk of thromboembolism whilst in ICU [5, 39], but optimal timing of anticoagulation is unknown [34, 52]. Two review articles [51, 56] proposed a patient-centred approach to only administer anticoagulants in patients with high risk of arterial thromboembolic events. Notably, 64% of respondents of a UK wide survey [17] reported that they would not use anticoagulant therapy in critically ill patients with NOAF.

Included studies were consistent in recommending further research as optimal management strategies have yet to be determined. Findings from previous studies of NOAF in patients in ICU may have been affected by the heterogeneity of patients in a general ICU. Future studies of narrower populations may therefore be helpful to determine best practice in specific clinical scenarios.

## Conclusions

Our systematic scoping review focusses on the comparative evidence for treatment of NOAF in patients in ICU. Interpretation of the evidence is limited due to study design flaws and important differences in definitions of NOAF, outcomes and treatment dose. Calcium channel blockers may result in more cardiovascular instability and slower rhythm control than amiodarone or beta blockers. More evidence is required about risk of bleeding and thromboembolism in the short and long term after NOAF onset. However, the little evidence available does not support therapeutic anticoagulation for NOAF whilst patients are critically ill. International guidelines regarding management of AF are largely based on studies and expert consensus that may not be applicable to this specific patient population. Given the significant morbidity and mortality associated with NOAF, adequately powered RCTs are needed to inform management of this common phenomenon. Consensus definitions of NOAF, and of treatment success will improve future studies.

## Supplementary Information

**Additional file 1.** Search strategy; detailed tables of studies by study design; critical appraisal; summary of reviews, surveys and opinion pieces; list of excluded studies.

## Data Availability

Data sharing is not applicable to this article as no datasets were generated or analysed during the current study.

## Abbreviations

AF: Atrial fibrillation
CCB: Calcium channel blockers
ICU: Intensive care unit
NOAF: New onset atrial fibrillation
RCT: Randomised controlled trial
