# Supplementary material for: Treatment strategies for new onset atrial fibrillation in patients treated on an intensive care unit: a systematic scoping review
Source: Crit Care. 2021 Jul 21;25:257. doi: 10.1186/s13054-021-03684-5 (PMC8296751; doi:10.1186/s13054-021-03684-5)
Supplement: Supplementary file 1 — Additional file 1. Search strategy; detailed tables of studies by study design; critical appraisal; summary of reviews, surveys and opinion pieces; list of excluded studies. [file 13054_2021_3684_MOESM1_ESM.docx]

Additional File

Contents

[1 Search strategy 3](#_Toc69725753)

[2 Detailed tables of studies by study design 8](#_Toc69725754)

[2.1 Randomized controlled trials 8](#_Toc69725755)

[2.2 Prospective comparative studies 12](#_Toc69725756)

[2.3 Retrospective comparative studies 16](#_Toc69725757)

[2.4 Prospective non-comparative studies 28](#_Toc69725758)

[2.5 Retrospective non-comparative studies 36](#_Toc69725759)

[3 Critical appraisal 47](#_Toc69725760)

[3.1 Risk of bias assessment for randomized trials 47](#_Toc69725761)

[3.2 Risk of bias assessment for large non-randomized studies 66](#_Toc69725762)

[4 Further summary tables 67](#_Toc69725763)

[5 Summary of reviews, surveys and opinion pieces 71](#_Toc69725764)

[5.1 Reviews and guidelines 71](#_Toc69725765)

[5.2 Systematic reviews 71](#_Toc69725766)

[5.3 Other types of review 72](#_Toc69725767)

[5.4 Surveys and opinion pieces 74](#_Toc69725768)

[5.4.1 Surveys 74](#_Toc69725769)

[5.4.2 Opinion pieces 74](#_Toc69725770)

[6 Excluded studies 76](#_Toc69725771)

[6.1 Excluded studies on full text with reason for exclusion 76](#_Toc69725772)

[6.2 List of excluded studies on full text 79](#_Toc69725773)

[7 References 90](#_Toc69725774)

# Search strategy

*MEDLINE*

Via Ovid; http://ovidsp.ovid.com/

1946 to present

Searched on: 4 March 2019

Records retrieved: 1087

Search strategy

1. ATRIAL FIBRILLATION/

2. ATRIAL FLUTTER/

3. SUPRAVENTRICULAR TACHYCARDIA/

4. ("atrial fibrillation*" or AF).ab,ti.

5. "atrial flutter* ".ab,ti.

6. "atrial arrhythmia* ".ab,ti.

7. ("supraventricular tachycardia*" or SVT).ab,ti.

8. "NOAF*".ab,ti.

9. 1 or 2 or 3 or 4 or 5 or 6 or 7 or 8

10. INTENSIVE CARE UNITS/

11. CRITICAL CARE/

12. SEPSIS/

13. SEPTIC SHOCK/

14. "intensive care".ab,ti.

15. "critical care unit* ".ab,ti.

16. "intensive therapy unit* ".ab,ti.

17. "high dependenc* ".ab,ti.

18. (ICU* or ITU* or HDU* or CCU*).ab,ti.

19. "critically unwell".ab,ti.

20. "critically ill".ab,ti.

21. (sepsis or "septic shock").ab,ti.

22. 10 or 11 or 13 or 14 or 15 or 16 or 17 or 18 or 19 or 20

23. 9 and 22

24. limit 23 to animals

25. limit 23 to ("newborn infant (birth to 1 month)" or "infant (1 to 23 months)" or "preschool child (2 to 5 years)" or "child (6 to 12 years)")

26. "case report* ".ti.

27. 24 or 25 or 26

28. 23 not 27

29. "atrial fibrillation* ".ab,ti.

30. (cardiac or cardiothoracic or "cardio thoracic" or cardiopulmonary or "cardio pulmonary").ab,ti.

31. "surg*".ab,ti.

32. "coronary care unit* ".ab,ti.

33. 29 and 30 and 31

34. 29 and 32

35. 33 or 34

36. 28 not 35

*EMBASE*

Via Ovid; <http://ovidsp.ovid.com/>

Searched on: 4 March 2019

Records retrieved: 3962

Search strategy

1. ATRIAL FIBRILLATION/

2. ATRIAL FLUTTER/

3. SUPRAVENTRICULAR TACHYCARDIA/

4. ("atrial fibrillation*" or AF).ab,ti.

5. "atrial flutter* ".ab,ti.

6. "atrial arrhythmia* ".ab,ti.

7. ("supraventricular tachycardia*" or SVT).ab,ti.

8. "NOAF*".ab,ti.

9. 1 or 2 or 3 or 4 or 5 or 6 or 7 or 8

10. INTENSIVE CARE UNITS/

11. CRITICAL CARE/

12. SEPSIS/

13. SEPTIC SHOCK/

14. "intensive care".ab,ti.

15. "critical care unit* ".ab,ti.

16. "intensive therapy unit* ".ab,ti.

17. "high dependenc* ".ab,ti.

18. (ICU* or ITU* or HDU* or CCU*).ab,ti.

19. "critically unwell".ab,ti.

20. "critically ill".ab,ti.

21. (sepsis or "septic shock").ab,ti.

22. 10 or 11 or 13 or 14 or 15 or 16 or 17 or 18 or 19 or 20

23. 9 and 22

24. limit 23 to animal studies

25. limit 23 to (infant <to one year> or preschool child <1 to 6 years> or school child <7 to 12 years>)

26. "case report* ".ti.

27. 24 or 25 or 26

28. 23 not 27

29. "atrial fibrillation* ".ab,ti.

30. (cardiac or cardiothoracic or "cardio thoracic" or cardiopulmonary or "cardio pulmonary").ab,ti.

31. "surg*".ab,ti.

32. "coronary care unit* ".ab,ti.

33. 29 and 30 and 31

34. 29 and 32

35. 33 or 34

36. 28 not 35

37. limit 36 to conference abstracts

38. 36 not 37

*CINAHL*

Searched on: 11 March 2019

Records retrieved: 441

1. CINAHL "ATRIAL FIBRILLATION"/ 20100

2. CINAHL "ATRIAL FLUTTER"/ 1521

3. CINAHL "TACHYCARDIA, SUPRAVENTRICULAR"/ 2593

4. CINAHL ("atrial fibrillation*" OR AF).ti,ab 24117

5. CINAHL ("atrial flutter*").ti,ab 1541

6. CINAHL ("atrial arrhythmia*").ti,ab 867

7. CINAHL ("supraventricular tachycardia*" OR SVT).ti,ab 1683

8. CINAHL (NOAF*).ti,ab 9

9. CINAHL (1 OR 2 OR 3 OR 4 OR 5 OR 6 OR 7 OR 8) 32813

10. CINAHL "INTENSIVE CARE UNITS"/ 32253

11. CINAHL "CRITICAL CARE"/ 19423

12. CINAHL "SHOCK, SEPTIC"/ 4198

13. CINAHL ("intensive care").ti,ab 50295

14. CINAHL ("critical care unit*").ti,ab 1967

15. CINAHL ("intensive therapy unit*").ti,ab 254

16. CINAHL ("high dependenc*").ti,ab 627

17. CINAHL (ICU* OR ITU* OR HDU* OR CCU*).ti,ab 24659

18. CINAHL ("critically unwell").ti,ab 17

19. CINAHL ("critically ill").ti,ab 17286

20. CINAHL (10 OR 11 OR 12 OR 13 OR 14 OR 15 OR 16 OR 17 OR 18 OR 19) 92933

21. CINAHL (9 AND 20) 480

22. CINAHL ("atrial fibrillation*").ti,ab 21936

23. CINAHL (cardiac OR cardiothoracic OR "cardio thoracic" OR cardiopulmonary OR "cardio pulmonary").ti,ab 114324

24. CINAHL (surg*).ti,ab 319271

25. CINAHL ("coronary care unit*").ti,ab 859

26. CINAHL ("case report*").ti 44822

27. CINAHL (22 AND 23 AND 24) 894

28. CINAHL (22 AND 25) 20

29. CINAHL (26 OR 27 OR 28) 45731

30. CINAHL 21 NOT 29 361

31. CINAHL 30 [Human age groups Infant~Newborn: birth-1 month OR Infant: 1-23 months OR Child~Preschool: 2-5 years OR Child: 6-12 years] 37

32. CINAHL "ANIMAL STUDIES"/ 98956

33. CINAHL (30 AND 32) 1

34. CINAHL (31 OR 33) 38

*Web of Science*

(includes Conference Proceedings Citation Index-Science, Science Citation Index Expanded, Social Sciences Citation Index, Arts & Humanities Citation Index, Conference Proceedings Citation Index-Science, Conference Proceedings Citation Index- Social Science & Humanities, The Book Citation Index-Science, The Book Citation Index- Social Science & Humanities, Emerging Sources Citation Index, Current chemical reactions-expanded, Index Chemicus)

1949 to 2019

Searched on: 6 March 2019

Records retrieved: 1772

Records retrieved after filtering by title: 137

Search strategy

“TOPIC: (atrial AND fibrillat*)

Refined by: TOPIC: ((intensive OR critical) AND (care OR therapy))

Indexes: SCI-EXPANDED, SSCI, A&HCI, CPCI-S, CPCI-SSH, BKCI-S, BKCI-SSH, ESCI, CCR-EXPANDED, IC”

*Cochrane library*

(includes Cochrane Reviews, Cochrane Protocols, Trials)

Searched on: 11 March 2019

Records retrieved in Cochrane Reviews: 0

Records retrieved in Cochrane Protocols: 0

Records retrieved in Trials: 96

Search strategy: "atrial fibrillation" AND ("intensive care" OR "critical care" OR “intensive therapy”)

*Database of Abstracts of Reviews of Effects (DARE).*

Searched on: 11 March 2019

Records retrieved: 4

Search strategy: (“atrial fibrillation” AND (“critical care” OR “intensive care”).

*OpenGrey*

Searched on: 11 March 2019

Records retrieved: 2

Search strategy: “Atrial fibrillation” AND (“Intensive Care” OR “Critical Care” OR “Intensive Therapy”)

*On-going, unpublished or grey literature search strategies*

*ClinicalTrials.gov*

URL: <https://clinicaltrials.gov/>.

Searched on: 11 March 2019

Records retrieved: 264

Search strategy: “Atrial fibrillation” in CONDITION field OR “Atrial flutter” in OTHER TERMS field

*ISRCTN*

Searched on: 11 March 2019

Records retrieved: 292

Search strategy: ATRIAL FIBRILLATION” in TEXT field

*EU clinical trials register*

Searched on: 11 March 2019

Records retrieved: 12

Search strategy: "atrial fibrillation" AND ("critical care" OR "intensive care" OR "intensive therapy")

*WHO ICTRP (World Health Organisation International Clinical Trials Registry Platform)*

Searched on: 11 March 2019

Records retrieved: 12

Search strategy: "atrial fibrillation" AND ("critical care" OR "intensive care" OR "intensive therapy")

*NIHR UK Clinical Trials Gateway*

Searched on: 11 March 2019

Records retrieved: 149

Search strategy: “atrial fibrillation”

# Detailed tables of studies by study design

## Randomized controlled trials

Supplementary table 1 - Methods and characteristics of randomized controlled trials

| Study details | Population characteristics | Intervention | Comparator |
| --- | --- | --- | --- |
| Authors  Balser et al. (1998)(1)  Setting  ICU  Country  USA  Sample size  n=55  n=28 (esmolol)  n=27 (diltiazem)  %NOAF patients  n=44 (80%)  n=22 (79%) (esmolol)  n=22 (81%) (diltiazem) | Primary diagnosis  Noncardiac surgical patients   \| Type of surgery \| Esmolol \| Diltiazem \| \| --- \| --- \| --- \| \| GI/GU, n \| 7 \| 13 \| \| Thoracic, n \| 9 \| 6 \| \| Nonthoracic vascular, n \| 4 \| 3 \| \| Neurosurgery, n \| 2 \| 3 \| \| Other, n \| 4 \| 2 \| \| No surgery, n \| 2 \| 0 \|   Mean age  66 ± 15 years (esmolol), 69 ± 11 years (diltiazem)  %Male  n=14 (50%) (esmolol), n=16 (59%) (diltiazem)  Severity of illness  APACHE III reported: 59 ± 31 (esmolol), 65 ± 24 (diltiazem)  %patients on vasopressors: n=1 (3.57%) (esmolol), n=3 (11%) (diltiazem)   \| %patients with CVD \| Esmolol \| Diltiazem \| \| --- \| --- \| --- \| \| Coronary artery disease, n (%) \| 10 (36%) \| 13 (48%) \| \| Recent MI or ischemia, n (%) \| 1 (3.57%) \| 2 (7%) \| \| Left ventricular hypertrophy, n (%) \| 3 (11%) \| 1 (3.7%) \|   %patients with acute renal failure: Not reported  %patients with acute respiratory failure: Not reported  Mechanical ventilation at NOAF onset: Not reported  Serum potassium: Not reported  Definition of NOAF: “SVT present for as long as 24 hours” | Esmolol  12.5 mg intravenous boluses initially, targeting heart rate <110 beats/min.  Followed by infusion titrated to maintain heart rates between 80 to 100 beats/min.  In the case of hypotension (SBP <80 mmHg or symptomatic), the infusion rate was decreased by 50% and/or a phenylephrine infusion was started.  Line of NOAF treatment:  Second line – adenosine given before the study treatment | Diltiazem  Loading dose of 20mg followed by a 10 mg/h infusion.  After 15 minutes, patients with heart rates >110 beats/min received an additional loading dose of 25 mg and infusion rate increased by 5mg/hr.  Infusion titrated to maintain heart rates between 80 and 100 beats/min.  In the case of hypotension (SBP <80 mmHg or symptomatic), the infusion rate was decreased by 50% and/or a phenylephrine infusion was started.  Line of NOAF treatment:  Second line – adenosine given before the study treatment |
| Authors  Delle Karth et al. (2001)(2)  Setting  ICU  Country  Austria  Sample size  n=60  n=20 (diltiazem)  n=20 (amiodarone bolus)  n=20 (amiodarone bolus + 24 hrs)  %NOAF patients  n=57 (95%) | Primary diagnosis   \|  \| Diltiazem \| Amiodarone Bolus \| Amiodarone bolus + 24 hrs \| \| --- \| --- \| --- \| --- \| \| Congestive heart failure, n \| 5 \| 4 \| 4 \| \| Coronary artery disease, n \| 2 \| 2 \| - \| \| Cardiac surgery, n \| 6 \| 9 \| 14 \| \| Respiratory failure, n \| 6 \| 2 \| 1 \| \| Others, n \| 1 \| 3 \| 1 \|   Mean age  64.8 ± 10 years (diltiazem), 67.8 ± 9 years (amiodarone bolus), 71.2 ± 9 years (amiodarone bolus + 24 hrs)  %Male  n=15 (75%) (diltiazem), n=17 (85%) (amiodarone bolus), n=11 (55%) (amiodarone bolus + 24 hrs)  Severity of illness  APACHE III score reported  75.1 ± 35 (diltiazem), 76.7 ± 38 (amiodarone bolus), 59.7 ± 8 (amiodarone bolus + 24 hrs)  %patients on vasopressors  At the time of onset  n=14 (70%) (diltiazem), n=15 (75%) (amiodarone bolus), n=15 (75%) (amiodarone bolus + 24 hrs)  %patients with CVD  n=13 (65%) (diltiazem), n=15 (75%) (amiodarone bolus), n=18 (90%) (amiodarone bolus + 24 hrs)  %patients with acute renal failure: Not reported  %patients with acute respiratory failure  n=6 (30%) (diltiazem), n=2 (10%) (amiodarone bolus), n=1 (5%) (amiodarone bolus + 24 hrs)  Mechanical ventilation at NOAF onset  n=15 (75%) (diltiazem), n=17 (85%) (amiodarone bolus), n=14 (70%) (amiodarone bolus + 24 hrs)  Serum potassium: Not reported  Definition of NOAF: Recent-onset tachycardic atrial fibrillation was defined as “atrial fibrillation with a rate consistently >120 beats/min over a 30-min period.” | Diltiazem  25 mg diltiazem by an intravenous bolus infusion over 15 mins followed by a continuous infusion at a rate of 20 mg/hr for a total of 24 hrs  Line of NOAF treatment  First line | Amiodarone bolus  A bolus dose of 300 mg amiodarone over 15 mins.  Line of NOAF treatment:  First line  Amiodarone bolus + 24 hrs  A bolus dose of 300 mg amiodarone over 15 mins followed by a continuous infusion at a rate of 45 mg/hr over 24 hrs.  Line of NOAF treatment  First line |

Supplementary table 2 - Results of randomized trials

| Authors | Results | Adverse effects | Recommendations/ barriers for future research |
| --- | --- | --- | --- |
| Balser et al. (1998)(1) | Fifty-nine percent of patients receiving esmolol converted to sinus rhythm within 2 h versus 33% of patients who received diltiazem (intention to treat; p = 0.049).  Conversion rates within 2 hours for patients with NOAF: 59% (esmolol group) versus 27% diltiazem group (p=0.067).  By 12 h, 85% of patients who received esmolol had converted to sinus rhythm compared with 62% of patients who received diltiazem (p=0.116). Length of ICU stay (days) between the groups: Esmolol: 8.4 ± 9.5 days versus diltiazem:  10.6 ± 13.4 days for diltiazem group; and did not find it to differ significantly.  In-hospital mortality was also reported not significantly different, 31% in esmolol group versus 38% in diltiazem group. | No adverse effects | “Although intuition suggests that ICU patients may benefit from accelerated conversion to sinus rhythm after operation, a much larger trial would be necessary to determine whether early conversion influences outcome” |
| Delle Karth et al. (2001)(2) | The number of patients achieving successful rate reduction (≥ 30%) within 4 hours was not significantly different between the groups – n=14 (70%) in diltiazem group (group 1) versus n=11 (55%) in amiodarone bolus group (group 2) versus n=15 (75%) in amiodarone bolus + 24 hrs group (group 3) (chi-square = 1.95; p = .38).  Conversion to sinus rhythm within 4 hours was not significantly different between the groups: n=6 (30%) group 1 versus n=8 (40%) group 2 versus n=9 (45%) group 3 (p=0.61). When the amiodarone groups were pooled, the occurrence of sinus rhythm was still not significantly different when compared to diltiazem group n=17/40 (42.5%) versus n=6/20 (30%), accordingly (chi-square = 0.88, p= .34)  Diltiazem showed slight but significantly better rate reduction when compared with amiodarone groups (F _group 1 vs. 3_ = 32.6, p = .0001; F _over time_ = 179, p = .0001; F _group 1 vs. 2_ = 48.7, p = .0001; F _over time_ = 117, p = .001). Authors reported no significant difference between the amiodarone groups (F _group 2 vs._ 3 = 3.02 p = .08, F _over time_ = 102.68, p = .0001). | Bradycardia developed in one patient in diltiazem group. Bradycardia was not observed in amiodarone groups. Hypotension resulting in discontinuation of the study drug was more common in the diltiazem group (n=6/20 [30%], n=0/20, n=1/20 [5%] for diltiazem, amiodarone bolus, and amiodarone bolus + 24 hours groups, respectively; p = .01). | Not reported |

## Prospective comparative studies

Supplementary table 3 - Methods and characteristics of prospective comparative studies (non-RCTs) of NOAF treatments

| Study details | Population characteristics | Intervention | Comparator |
| --- | --- | --- | --- |
| Authors  Gerlach et al. (2008)(3)  Setting  Surgical ICU  Country  USA  Sample size  n=61  %NOAF patients  n=55 (90%)  n=28 (diltiazem)  n=27 (amiodarone) | Primary diagnosis  Mixed population:   \|  \| Diltiazem \| Amiodarone \| \| --- \| --- \| --- \| \| Trauma, n \| 5 \| 9 \| \| Gastrointestinal, n \| 15 \| 11 \| \| Vascular, n \| 4 \| 5 \| \| Other surgery, n \| 7 \| 5 \|   Mean age  Mixed population:  68.5±14.6 years (diltiazem group), 66.1±16 years (amiodarone group)  % Male  Mixed population:  n=18 (58%) (diltiazem), n=21 (70%) (amiodarone)  Severity of illness: Not reported  %patients on vasopressors  At the time of onset  n=11 (18%), n=8 (26%) (diltiazem) , n=3 (10%) (amiodarone)  %patients with CVD  n=11 (18%), n=5 (16%) (diltiazem) , n=6 (20%) (amiodarone)  % patients with acute renal failure: Not reported  % patients with acute respiratory failure: Not reported  Mechanical ventilation at NOAF onset: Not reported  Serum potassium: Not reported  Definition of NOAF: Not reported | Intervention treatment given to patients during the first year of the study  Diltiazem 0.25 mg/kg IV bolus, followed by infusion of 5-15 mg/hr titrated to target heart rate less <120 beats per minute.  After 48 hours, the infusion was continued, discontinued, or switched to oral therapy at the discretion of the treating clinicians.  Line of NOAF treatment  Not specifically reported; however no other treatments reported | Comparator treatment given to patients during the second year of the study  Amiodarone 150 mg intravenously, over at least 10 minutes, followed by infusion of 1 mg/min for 6h then decreased to 0.5mg/min. After 48 hours, the infusion was continued, discontinued, or switched to oral therapy at the discretion of the treating clinicians.  Line of NOAF treatment  Not specifically reported; however no other treatments reported |
| Authors  Launey et al. (2019)(4)  Setting  5 academic ICUs  Country  France  Sample size  n=261  n=123 (hydrocortisone)  n=138 (no hydrocortisone)  %NOAF patients  Not applicable | Primary diagnosis: Septic shock   \| Origin of septic shock \| HC^[[1]](#footnote-2)^ \| No HC \| \| --- \| --- \| --- \| \| Intra-abdominal, n (%) \| 72 (59) \| 72 (52) \| \| Thoracic, (%) \| 24 (20) \| 32 (23) \| \| Urinary, (%) \| 17 (14) \| 14 (10) \| \| Other, (%) \| 10 (8) \| 20 (14) \|   Mean age  65 ± 13 years (HC), 63 ± 15 years (no HC)  %Male  61% (HC), 58% (no HC)  Severity of illness  Mean SOFA score (baseline)  10 ± 4 (HC), 8 ± 3 (no HC)  Mean SAPS II score (baseline)  56 ± 20 (HC), 50 ± 20 (no HC)  Mean SOFA score reported (during the first 24 hours of septic shock)  13 ± 0 (HC), 10 ± 0 (no HC)  %patients on vasopressors   \|  \| HC \| No HC \| \| --- \| --- \| --- \| \| Norepinephrine, n (%) \| 122 (99) \| 135 (98) \| \| Dobutamine, n (%) \| 29 (24) \| 6 (4) \| \| Epinephrine, (%) \| 15 (12) \| 10 (7) \|   %patients with CVD   \|  \| HC \| No HC \| \| --- \| --- \| --- \| \| Coronary disease, n (%) \| 11 (9) \| 12 (9) \| \| Valvular disease, n (%) \| 5 (4) \| 7 (5) \|   %patients with acute renal failure: Not reported  %patients with acute respiratory failure: Not reported  Mechanical ventilation at NOAF onset: Not reported  Serum potassium: Not reported  Definition of NOAF: Atrial fibrillation was defined as chaotic atrial activity with no apparent P waves and irregular ventricular activity lasting at least 30 seconds | A hydrocortisone bolus of 100mg followed by a infusion of 200mg/day for 7 days followed by a short wean if the patient remained on vasopressors.  Line of NOAF treatment  Not applicable as prophylactic treatment studied | No prophylactic treatment |

Supplementary table 4 - Results of prospective comparative studies (non-RCTs) of NOAF treatments

| Authors | Methods to address confounding | Results | Adverse effects | Recommendations/ barriers for future research |
| --- | --- | --- | --- | --- |
| Gerlach et al. (2008)(3) | None reported | No differences between treatments.  24 hours conversion rate (%) 87.1 (diltiazem) and 86.7 (amiodarone) p = 0.96. Mean time to conversion (hours) 6.9 (diltiazem) and 5 (amiodarone) p = 0.35  Both groups had similar lengths of ICU stay (mean days ± SD) (13.5±11.9 days in the diltiazem group versus 11.6±10.9 days in the amiodarone group, p =0.54) and hospital length of stay (22.5±18.9 days in the diltiazem group compared to 24.2±24.2 in the amiodarone group, p =0.76) | Transient hypotension  (diltiazem n=1, amiodarone n=2). In the diltiazem patient, hypotension resolved after decreasing the infusion rate. For the amiodarone recipients, one was treated with fluids, and the other’s blood pressure recovered spontaneously. | “More studies including randomized controlled trials are needed to compare the use of diltiazem versus amiodarone for conversion of post- operative AF” |
| Launey et al. (2019)(4) | The authors estimated propensity scores using a logistic regression in which treatment status (the administration of low- dose hydrocortisone) was regressed on the variables that were related to the outcome (the occurrence of AF) in the univariate analyses (P < 0.20). The inverse probability of treatment weighting (IPTW) method was then used to assign weights to the samples according to the propensity scores.  The authors performed an additional analysis in which multivariate logistic regression was done with the same set of covariates that were used for the IPTW analysis (age; chronic renal failure and thyroid disorder; SAPS II and SOFA scores acquired at admission; cardiac surgery during the present hospitalization; corticotherapy before the onset of septic shock; dobutamine and epinephrine use during septic shock; maximum dose of catecholamine(s) during septic shock (norepinephrine, epinephrine); white blood cell count during septic shock; the presence of a central venous catheter, pulmonary artery catheter, or mediastinal drainage during septic shock; and dialysis during septic shock). | AF was diagnosed in 57 (22%) of the patients. The AF rates were 33 (24%) and 24 (19%) in the no-HC and HC groups, respectively. In the weighted sample, the proportions of patients who developed AF were 28.8% and 16.8% in the no-HC and HC groups, respectively. The risk difference was -11.9% (95%CI: -23.4% to -0.5%, P = .040) and the relative risk was 0.58 (95% CI: 0.35-0.98, P= .041). In a multivariate logistic regression model, the estimated marginal effect was -12.6% (95% CI: -22.3% to -2.5%, P = .013)  Similar ICU length of stays (12 ± 1 days in HC group versus 13 ± 1 days in no HC group, P = .730) and hospital length of stays (34 ± 3 days in HC group versus 32 ± 3 days in no HC group, P = .560)  ICU mortality was reported as 46 (37%) in HC group versus 33 (24%) in no HC group, P = .018. 28-day mortality was reported as 47 (38%) in HC group versus 36 (26%) in no HC group, P = .036. | None reported | ‘It would be interesting to study high-risk patients who develop AF and the short- and long-term outcomes in patients treated or not with hydrocortisone.’ |

## Retrospective comparative studies

Supplementary table 5 - Methods and characteristics of retrospective comparative studies of NOAF treatments

| Study details | Population characteristics | Intervention | Comparator |
| --- | --- | --- | --- |
| Authors  Balik et al. (2017)(5)  Setting  15 bed general ICU  Country  The Czech Republic  Sample size  n=234  n=177 (amiodarone)  n=42 (propafenone)  n=15 (metoprolol)  %NOAF patients  n = 163 (69.7%) | Primary diagnosis: Septic shock  Primary sources of septic shock:  Respiratory 57.3%, Abdominal 25.2%, Urosepsis 7.3%, Wound/surgical 5.2%, Catheter related 4.2%, Maxilofacial 0.4%, Neuroinfection 0.4%  Mean age  67.8 ± 11.4 (amiodarone), 66.8 ± 11.3 (propafenone), 60.9 ± 8.3 (metoprolol)  %Male: n=139 (59.4%)  Severity of illness  At the start of the antiarrhythmic therapy  APACHE II  25 ± 11.4 (amiodarone), 23.2 ± 11.1 (propafenone), 19.4 ± 11.9 (metoprolol)  SOFA  11.1 ± 4 (amiodarone), 10.2 ± 4 (propafenone), 7.0 ± 4.2 (metoprolol)  % patients on vasopressors   \|  \| Amiodarone \| Propafenone \| Metoprolol \| \| --- \| --- \| --- \| --- \| \| Dobutamine, n (%) \| 24 (16.9) \| 6 (7.7) \| - \| \| Vasopressin, n (%) \| 10 (7) \| 2 (2.6) \| - \|   % patients with CVD  n=117 (50%)  n=79 (56%) (amiodarone) , n=34 (43.6%) (propafenone) , n=4 (28.6%) (metoprolol)  % patients with acute renal failure: n=64 (27.4%)  % patients with acute respiratory failure: Not reported  Mechanical ventilation at NOAF onset: n=232 (99.1%); though does not specify whether this was at the onset  Serum potassium (mmol/L)  4.4 ± 0.6 (amiodarone), 4.4 ± 0.6 (propafenone), 4.3 ± 0.5 (metoprolol)  Definition of NOAF: Not reported | Propafenone  The median total dose of propafenone 2.5g (IQR 1.0–4.0). The length of therapy was 5.0 days (2.0–8.5)  Line of NOAF treatment  First and second line | Amiodarone  Median total dose of amiodarone was 3.0g (1.8–4.6), given by infusion over 4 (2–6) days  Line of NOAF treatment  First and second line  Metoprolol  The median IV metoprolol dose was 84mg/day (48–120).  The median length of therapy was 5 days (2-9)  Line of NOAF treatment  First line |
| Authors  Cho et al. (2017)(6) (conference abstract)  Setting  Medical ICU  Country  South Korea  Sample size  n=448  %NOAF patients  100% | Primary diagnosis: Sepsis  Mean age: 68.2 years  %Male: 68.9%  Severity of illness  Median CHADS2VASC score: 3, Median APACHE II score: 24  %patients on vasopressors: 59.9% (at the time of NOAF onset)  %patients with CVD: Not reported  %patients with acute renal failure: Not reported  %patients with acute respiratory failure: Not reported  Mechanical ventilation at NOAF onset: 84.5%  Serum potassium: Not reported  Definition of NOAF: Not reported | Rhythm control (43.5% patients)  Amiodarone used in 95.4% of rhythm control cohort  Line of NOAF treatment:  Not specified | Rate control (56.7% patients)  Line of NOAF treatment:  Not specified |
| Authors  Jaffer et al. (2016)(7) (conference abstract)  Setting  ICU  Country  USA  Sample size  n=65  %NOAF patients  100% | Primary diagnosis: Septic shock  Mean age: Not reported  %Male: 56%  Severity of illness: Not reported  %patients on vasopressors: Not reported  %patients with CVD: Not reported  %patients with acute renal failure: Not reported  %patients with acute respiratory failure: Not reported  Mechanical ventilation at NOAF onset: Not reported  Serum potassium: Not reported  Definition of NOAF: Not reported | Amiodarone (administered to 49% of patients)  Line of NOAF treatment:  Not specified | Calcium channel blockers (administered to 15% of patients)  Beta blockers (administered to 12% of patients)  Line of NOAF treatment:  Not specified |
| Authors  Kane and Hanes (2014)(8) (conference abstract)  Setting  ICU  Country  USA  Sample size  n=109  n=39 (hydrocortisone)  %NOAF patients  Not applicable as prophylactic treatment studied | Primary diagnosis: Septic shock  Mean age: Not reported  %Male: Not reported  Severity of illness  mean APACHE IV score reported: 97 ± 32.5  %patients on vasopressors: Not reported  %patients with CVD: Not reported  %patients with acute renal failure: Not reported  %patients with acute respiratory failure: Not reported  Mechanical ventilation at NOAF onset: Not reported  Serum potassium: Not reported  Definition of NOAF: Not reported | Hydrocortisone (median duration of 4.2 days (IQR 1.1–8.1).  Line of NOAF treatment:  Not applicable as prophylactic treatment studied | No treatment  Line of NOAF treatment:  Not applicable |
| Authors  Matsumoto et al. (2015)(9) (conference abstract)  Setting  ICU  Country  Japan  Sample size  n=276  n=116 (amiodarone)  n=160 (landiolol)  %NOAF patients: 100% | Primary diagnosis: Not reported  Mean age: Not reported  %Male: Not reported  Severity of illness: Not reported  %patients on vasopressors: Not reported  %patients with CVD: Not reported  %patients with acute renal failure: Not reported  %patients with acute respiratory failure: Not reported  Mechanical ventilation at NOAF onset: Not reported  Serum potassium: Not reported  Definition of NOAF: Not reported | Amiodarone  A loading dose of 150mg over 30 mins followed by a continuous infusion of 20 mg/h.  Line of NOAF treatment:  Not specified | Landiolol  A bolus dose of 7.5mg followed by continuous infusion of 2.5 - 7.5 mg/h.  Line of NOAF treatment:  Not specified |
| Authors  McKenzie Brown et al. (2018)(10)  Setting  Surgical Intensive Care Unit  Country  USA  Sample size  n=33  Initially treated with:  β-blockers n=22  amiodarone n=6  calcium channel blockers n=2  no treatment n=3  %NOAF patients  100%  NOAF with rapid ventricular rate (RVR) | Primary diagnosis  Esophagectomy n=8, Intra-abdominal surgery n=9, Other surgery n=9, Trauma n=7  Sepsis at the time of onset (n=16 (48.5%))  Mean age: Median age (IQR) = 71 (64, 80) years  %Male: n=19 (58)  Severity of illness: not reported for baseline or onset characteristics  %patients on vasopressors: (within 24 hours of NOAF onset): n=12 (36%)  %patients with CVD  Coronary artery disease 20%, Stroke 12%, Peripheral vascular disease 9%  %patients with acute renal failure: Not reported  %patients with acute respiratory failure: Not reported  Mechanical ventilation at NOAF onset:  Only reported for within 24 hours of onset: n=5 (15%)  Serum potassium  Patients with K < 4 mmol/L on first laboratory after AF onset: n= 15 (45%)  Definition of NOAF: “New-onset post-operative atrial fibrillation was defined as development of AF in any patient with no prior history of AF as documented in the patient history or review of available medical records. AF with RVR was defined as a heart rate >100 beats per minute based on the highest recorded rate during the period of AF” | β-blockers  Line of NOAF treatment  First line | Amiodarone  Calcium channel blockers  Line of NOAF treatment  First, second and third  Sixteen patients (48%) received a second medication due to failure to restore sinus rhythm, with amiodarone being the most common (n = 13 (81%)). |
| Authors  Mieure et al. (2011) (conference abstract)(11)  Setting  ICU  Country  USA  Sample size  n=126  n=61 (amiodarone)  n=41 (diltiazem)  n=24 (metoprolol)  %NOAF patients: 100% | Primary diagnosis: Not reported  Mean age: Not reported  %Male: Not reported  Severity of illness: Not reported  %patients on vasopressors: Not reported  %patients with CVD: Not reported  %patients with acute renal failure: Not reported  %patients with acute respiratory failure: Not reported  Mechanical ventilation at NOAF onset: Not reported  Serum potassium: Not reported  Definition of NOAF  “onset 120 beats per minute, bpm” | Amiodarone  Line of NOAF treatment  Not specified | Diltiazem  Line of NOAF treatment  Not specified  Metoprolol  Line of NOAF treatment  Not specified |
| Authors  Walkey et al. (2016)(12)  Setting  20% of hospitals in the USA  Country  USA  Sample size  n=39,693  n=14,202 (calcium channel blockers (CCB))  n=11,290 (β-blockers)  n=7,937 (digoxin)  n=6,264 (amiodarone)  %NOAF  n= 3,174  Note: outcomes reported separately on NOAF patients | Primary diagnosis: Sepsis  Infection site, n (%)   \|  \| β-blocker \| CCB \| Digoxin \| Amiodarone \| \| --- \| --- \| --- \| --- \| --- \| \| Pneumonia \| 3,583 (31.7) \| 5,882 (41.4) \| 3,118 (39.3) \| 2,369 (37.8) \| \| GI \| 2,107 (18.7) \| 1,692 (11.9) \| 1,030 (13.0) \| 896 (14.3) \| \| Urinary tract \| 4,173 (37.0) \| 5,439 (38.3) \| 3,008 (37.9) \| 1,980 (31.6) \| \| Skin or soft tissue \| 982 (8.7) \| 1,217 (8.6) \| 696 (8.8) \| 507 (8.1) \| \| Primary bacteraemia or fungemia \| 140 (1.2) \| 150 (1.1) \| 82 (1.0) \| 76 (1.2) \|   Mean age  75.7 ± 11.3 years (β-blocker), 75.6 ± 11.4 years (CCB), 77.1 ± 10.7 years (digoxin), 73.1 ± 11.7 years (amiodarone)  %Male  50.4% (β-blocker), 47.4% (CCB), 48.5% (digoxin), 55.1% (amiodarone)  Severity of illness: Not reported  %patients on vasopressors  On first hospital day  β-blockers 29.1%, CCB 26.5%, Digoxin 44.1%, Amiodarone 64.0%  %patients with CVD: Not reported  %patients with acute renal failure: Not reported  %patients with acute respiratory failure: Not reported  Mechanical ventilation at NOAF onset: Not reported  Serum potassium: Not reported  Definition of NOAF: if AF was not present on admission | IV β-blocker (metoprolol, esmolol, atenolol, labetalol,  propranolol)  Line of NOAF treatment:  Not specified | IV Calcium channel blocker (diltiazem, verapamil)  IV Digoxin  IV Amiodarone  Line of NOAF treatment:  Not specified |
| Authors  Walkey et al. (2016)b(13)  Setting  Non-federal US hospitals  Country  USA  Sample size  n= 38,582  n=31,060 (pre-existing AF)  %NOAF  n=7522  n=5585 analysed with propensity score approach  Note: outcomes reported separately on NOAF patients | Primary diagnosis: Sepsis  Primary infection site, n (%)   \|  \| Anticoagulation, n=13 611 (35.3%) \| No anticoagulation  n=24 971 (64.7%) \| \| --- \| --- \| --- \| \| Pneumonia \| 5537 (40.7) \| 9012 (36.1) \| \| Gastrointestinal tract infection \| 2040 (15.0) \| 3892 (15.6) \| \| Urinary tract infection \| 4550 (33.4) \| 9194 (36.8) \| \| Skin or soft-tissue infection \| 1315 (9.7) \| 1766 (7.1) \| \| Primary bacteremia or fungemia \| 153 (1.1) \| 305 (1.2) \|   Mean age  73.2 ± 11.7 years (anticoagulation), 75.8 ± 11.7 years (no anticoagulation)  %Male  n=6941 (51%) (anticoagulation), n=12,035 (42.2%) (no anticoagulation)  Severity of illness  mean (SD) CHA2DS2VASc score reported  3.4 (1.5) (anticoagulation), 3.6 (1.5) (no anticoagulation)  %patients on vasopressors  n=5084 (37.4%) (anticoagulation), n=10 002 (40.1%) (no anticoagulation)  %patients with CVD   \|  \| Anticoagulation,  n=13 611 (35.3%) \| No anticoagulation, n=24 971 (64.7%) \| \| --- \| --- \| --- \| \| Heart failure, n (%) \| 5712 (42.0) \| 9792 (39.2) \| \| Coronary heart disease or myocardial infarction, n (%) \| 4532 (33.3) \| 7970 (31.9) \| \| Valvular heart disease, n (%) \| 2010 (14.8) \| 3348 (13.4) \|   %patients with acute renal failure  acute kidney failure reported  n= 7612 (55.9%) (anticoagulation), n= 15 814 (63.3%) (no anticoagulation)  %patients with acute respiratory failure  n= 5308 (39.0%) (anticoagulation), n= 9442 (37.8%) (no anticoagulation)  Mechanical ventilation at NOAF onset: Not reported  Serum potassium: Not reported  Definition of NOAF: “incident AF that was not present on admission” | Intravenous  or subcutaneous administration of therapeutic-dose anticoagulant.  Patients who received oral anticoagulants as their initial anticoagulant were excluded in the primary analysis.  Line of NOAF treatment:  Not applicable | No anticoagulation  Line of NOAF treatment:  Not applicable |

Supplementary table 6 - Results of retrospective comparative studies of NOAF treatments

| Authors | Methods to address confounding | Results | Recommendations/ barriers for the future research |
| --- | --- | --- | --- |
| Balik et al. (2017)(5) | Linear regression analysis involving univariate and multivariate testing | Amiodarone  Restoration to sinus rhythm was observed in 74% patients in amiodarone group. Four patients were switched to amiodarone group from propafenone group due to failure to restore sinus rhythm and one patient from the metoprolol group due to haemodynamic instability. Cardioversion was achieved in 114 patients. However, 23.7% of those required additional electric cardioversion. Forty patients (26%) who failed to restore sinus rhythm were switched to propafenone group during the first 24 hours.  Propafenone  Sinus rhythm was achieved in 88.9%. Forty patients were moved from amiodarone to propafenone. Four patients were switched from propafenone to amiodarone. Overall cardioversion success rate of 86.1% at 24 h was reported. Of those, 35.5% needed additional electric cardioversion to achieve sinus rhythm.  Metoprolol  Sinus rhythm was restored in 92.3% patients without any additional electric cardioversion. One patient was moved to amiodarone infusion.  Authors reported statistically not significant but higher ICU mortality of amiodarone (40.4%, OR=1.79) compared to 30.4% of propafenone and 21.4% of metoprolol. Twenty-eight-day mortality was reported highest in amiodarone group (49.6%) compared to propafenone group (39.5%) and metoprolol group (21.4%). In the univariate one-year survival analysis, long term survival of propafenone group was similar to long term survival of metoprolol group. Long term survival in both propafenone and metoprolol groups was significantly higher than in amiodarone group (HR=1.76, 95%CI (1.06; 2.3), p = 0.02). The authors confirmed the result by multivariate survival analysis. This was corrected for age, dosage of noradrenaline, SOFA score and rate of CRRT (HR=1.58, 95%CI (1.04; 2.4), p = 0.03). In the univariate analysis which excluded chronic AF patients, a one-year mortality benefit in favour of restoration of sinus rhythm in septic shock (HR=0.48, p=0.002) was showed. After adjustment for age, dosage of noradrenaline, SOFA score and presence of CRRT, the result was not statistically significant. (HR=0.67, p = 0.113). | Not reported |
| Choi et al. (2017) (abstract)(6) | Propensity matching | The authors reported that patients managed by rhythm control strategy showed higher sinus conversion rate compared to those with rate control strategy (39.8% vs. 19.8%, p<0.001). However, mortality rate (54.9% vs. 49.3%, p=0.529) or thromboembolic events (5.5% vs. 7.6%, p=0.635) did not differ between the two groups. | Not reported |
| Jaffer et al. (2016) (abstract)(7) | None | The authors reported no significant difference between the groups in analysis of the primary outcome of mortality or for the secondary outcomes of length of ICU and hospital stay. The MELD score (liver function) at end of ICU for the amiodarone group (20.85 +/- 8.70) was significantly higher (p-value 0.03) when adjusted for age and gender compared to the control (15.40), BB (12.88) and CCB (17.10) groups. | “Further investigation into the management of arrhythmias in septic shock is needed to further elucidate the potential benefits and harms of various pharmaceutical agents.” |
| Kane and Hanes (2014) (abstract)(8) | Multivariate regression | The authors reported the overall incidence of AF as 34.9%. It was concluded that NOAF was significantly less common in patients  who received hydrocortisone than those who did not (20.5% vs. 42.9%, p=0.022). The authors reported that multivariate regression showed the receipt of hydrocortisone was significantly associated with a reduction in NOAF (p=0.006). No difference in mortality, AF requiring intervention, or length of stay was reported. | “Given the incidence rate of AF in septic shock, a preventative study using HC may be appropriate. Furthermore, future studies using HC in this patient population should include AF as a secondary endpoint.” |
| Matsumoto et al. (2015) (9) (abstract) | Methods not reported | The authors reported that single drug pharmacological cardioversion was attempted with amiodarone in 26 cases (50% cardioversion rate).Single drug pharmacological cardioversion with landiolol was attempted in 42 cases (67% cardioversion rate).  The mean time to sinus conversion:  124 minutes (95% CI 66-182) (amiodarone)  72 minutes (95% CI 52-91) (landiolol) No evidence of difference was found for sinus conversion rates between amiodarone and landiolol groups. The authors reported that patients receiving landiolol had statistically significant faster sinus rhythm recovery compared to those who received amiodarone. (p<0.001) | Not reported |
| McKenzie Brown et al. (2018) (10) | Markov chain analysis to account for patients who have achieved the outcome (sinus rhythm restoration). This analysis recognises that the outcome can be achieved by different medication.  No other methods to address confounding reported. | Authors reported that amiodarone was most successful achieving the rate and rhythm control in both cases, as an initial treatment and as a second line treatment – 6 patients (27%) who received β-blockers as a first line therapy converted to sinus rhythm versus 5 patients (83%) who received amiodarone as a first line treatment versus 1 patient (50%) who received calcium channel blocker as a first line treatment; 11 patients (85%) who received amiodarone as a second treatment converted to sinus rhythm versus 1 patients (33%) who received calcium channel blockers as a second line treatment converted to sinus rhythm. Markov chains analysis showed that administering amiodarone as a first, second, or third line medication was more likely to result in rate and rhythm control than if b-blockers were administered (P = 0.001).  The greatest success rate (92%) to convert to sinus rhythm was when beta blockers were used first, followed by amiodarone. This may suggest an additive effect of the two medications. | “Future studies are needed to further explore this and determine many unknowns including optimal dosing and route, need for anticoagulation, and duration of treatment” |
| Mieure et al. (2011) (abstract) (11) | Methods not reported | The ventricular rate control (VR) to <100 bpm within 24 hours from initiation of treatment was achieved in 85.2% (52/61) of amiodarone patients, 85.0% (35/41) of diltiazem patients, and 87.5% (21/24) of metoprolol patients (P=1.00). The authors reported that the mean relative heart rate reduction (±SD) was 40.5±13%, 38±16%, and 41.9±12%, in amiodarone, diltiazem, and metoprolol groups, respectively (P=0.52). Conversion to and maintenance of sinus rhythm throughout the study period occurred in 21.3% of amiodarone patients, 7.3% of diltiazem patients, and 37.5% of metoprolol patients (P=0.013). | “A large randomized controlled trial designed to determine the optimal therapeutic strategy for a heterogeneous cohort of patients with new onset AF with RVR is needed.” |
| Walkey et al. (2016) (12) | Propensity score matching approach using over 30 covariates covering specific patient demographics, hospital characteristics, prevalent comorbidities, type of acute organ failure, and type of infection | Beta-Blockers versus CCBs  No differences in hospital mortality between the groups; RR 0.99 (95% CI: 0.86-1.15)  Beta-Blockers versus digoxin  RR of hospital mortality for patients who received beta-blockers compared to patients who received digoxin was 0.75 (95% CI: 0.64-0.88), indicating a better outcome for patients treated with beta-blockers.  Beta-Blockers versus amiodarone  RR of hospital mortality for patients who received beta-blockers compared to patients who received amiodarone was 0.67 (95% CI: 0.59-0.77), indicating a better outcome for patients treated with beta-blockers. | “Our outcome findings should be considered hypothesis generating and supportive of the need for future clinical trials to investigate optimal treatment of AF during sepsis” |
| Walkey et al. (2016)b (13) | A propensity score approach was used to adjust for variables representing hospital characteristics, patient demographics, comorbidities, use of intensive care, measures of acute organ dysfunction, source of infection, and year of hospitalization. | Authors reported relative risk (RR) of in-hospital ischemic stroke associated with anticoagulation as 0.85; 95% CI (0.57-1.27) for patients with newly diagnosed AF. The RR of bleeding associated with parenteral anticoagulation was reported as 0.97; 95%CI (0.83-1.14) for patients with newly diagnosed AF. | “Whereas current evidence suggests that benefits may not outweigh risks of parenteral anticoagulation for AF during sepsis, further study is warranted to determine optimal timing for restarting treatment with oral anticoagulants among patients with pre-existing AF and long-term anticoagulation strategies after hospitalization for patients with newly diagnosed AF during sepsis.” |

## Prospective non-comparative studies

Supplementary table 7 - Methods and characteristics of prospective single group studies

| Study details | Population characteristics | Intervention |
| --- | --- | --- |
| Authors  Delle Karth et al. (2005)(14)  Setting  Cardiologic ICU  Note: medical patients included in the study  Country  Austria  Sample size  n=17 (data extracted only for medical patients; the study also includes cardiac-surgical patients)  %NOAF patients  n=15 (88.2%) | Primary diagnosis  Respiratory failure 17.6%, Heart failure 35%, Sepsis 17.6%, Cardiopulmonary resuscitation 11.7%  Mean age  Median (range) age reported: 63 (56.5–73) years  %Male: n=11 (64.7%)  Severity of illness  Median (range) SAPS II score reported: 53 (44.5–63)  %patients on vasopressors: n=15 (88.2%)  %patients with CVD: n=6 (35%) (heart failure)  %patients with acute renal failure: n=3 (17.6%)  %patients with acute respiratory failure: Not reported  Mechanical ventilation at NOAF onset: n=13 (76.5%)  Serum potassium: Not reported  Definition of NOAF  “recent-onset (< 1 h) tachycardic (> 100 beats per min) sustained (> 10 min) atrial fibrillation” | Ibutilide  A 10-minute IV infusion of 1mg ibutilide. If AF did not terminate within 10min of the first infusion, a second 1mg infusion was given. If AF remained after 1h, ibutilide therapy was considered unsuccessful and further treatment was started at the discretion of the treating clinician.  Line of NOAF treatment:  Not specified |
| Authors  Hennersdorf et al. (2002)(15)  Setting  University hospital ICU  Country  Germany  Sample size  n=26  %NOAF patients  n=7 (27%)  Note: results reported separately for NOAF group | Primary diagnosis  Hypertension 31%, hypertrophic obstructive cardiomyopathy 8%, dilative cardiomyopathy 12%, Cor pulmonale 15%, coronary artery disease 19%, sepsis with cardiac involvement 15%, reduced left ventricular ejection fraction 15%  Mean age: 68 ± 11 years  %Male: n= 21 (80.7%)  Severity of illness  APACHE II score, median (range): 8 (5–24)  %patients on vasopressors: Not reported  %patients with CVD  Hypertrophic obstructive cardiomyopathy 8%, dilative cardiomyopathy 12%, Cor pulmonale 15%, coronary artery disease 19%, reduced left ventricular ejection fraction 15%  %patients with acute renal failure: Not reported  %patients with acute respiratory failure: Not reported  Mechanical ventilation at NOAF onset: n=19 (73%) (not specified if at the onset)  Serum potassium: Not reported  Definition of NOAF: Not reported | A 10-minute IV infusion of 1mg ibutilide. If AF did not terminate within 10min of the first infusion, a second 1mg infusion was given.  Prior to ibutilide, all patients were given IV magnesium 1g and IV potassium if serum concentration <4.5 mmol/l).  Line of NOAF treatment:  Second line  All patients received 150mg IV amiodarone as the first line drug. If patients failed to convert back to sinus rhythm, ibutilide was given intravenously 2 h after the administration of amiodarone. |
| Authors  Mayr et al. (2003)(16)  Setting  Surgical ICU  Country  Austria  Sample size  n=37  %NOAF patients  n=31 (84%) | Primary diagnosis  Surgery type: Abdominal 35.1%, cardiac 35.1%, vascular 2.7%, trauma 10.81%, orthopaedic 8.1%, thoracic 5.4%  Pneumonia after surgery 2.7%  Mean age  Median years (range): 72 (34–94) (all patients), 67 (34–82) (primary responders), 73.5 (51–94) (non-responders)  %Male  n=16/37 (43.2%) (all patients), n=7/13 (53.8%) (primary responders), n=9/24 (37.5%) (non-responders)  Severity of illness  Median (range) SAPS score reported: 13 (5–29) (all patients), 14 (5–29) (primary responders), 13 (9–26) (non-responders)  %patients on vasopressors  n=29/37 (78.37%) (all patients), n=10/13 (76.9%) (primary responders), n=19/24 (79.16%) (non-responders)  %patients with CVD   \|  \| All patients (n=37) \| Primary responders (n=13) \| Non-responders (n=24) \| \| --- \| --- \| --- \| --- \| \| Coronary artery disease, n (%) \| 22 (59.45) \| 7 (53.8) \| 15 (62.5) \| \| Heart failure, n (%) \| 13 (35) \| 4 (30.76) \| 9 (37.5) \|   %patients with acute renal failure: Not reported  %patients with acute respiratory failure: Not reported  Mechanical ventilation at NOAF onset: Not reported  Serum potassium (mmol/L)  4.2 (3.5–5) (all patients), 4.2 (3.5–5) (primary responders), 4.2 (3.6–4.7) (non-responders)  Definition of NOAF  “SVT were defined as narrow-complex, non-sinus tachycardias with heart rate >100 beats/min for at least 15 mins” | Electrical cardioversion.  If required, patients were sedated with etomidate.  A maximum of four consecutive shocks were administered.    In patients not responding to electrical cardioversion or in recurrent arrhythmia, intravenous antiarrhythmic therapy was started.  Line of NOAF treatment:  Not specified |
| Authors  Nakamura et al. (2016)(17)  Setting  Medical/surgical ICU for in-hospital patients and emergency ICU for emergency outpatients  Country  Japan  Sample size  n=16  %NOAF patients  100% | Primary diagnosis  Sepsis 50%, Heart failure 56.2%  Mean age: 75.0 ± 13.1 years  %Male: n=7 (43.75%)  Severity of illness  Mean APACHE II score reported: 24.3 ± 6.0  Mean SOFA score reported: 8.6 ± 3.1  %patients on vasopressors: In 62.5% of patients, noradrenaline had been given with landiolol  %patients with CVD: n=9 (56.2 %) (heart failure)  %patients with acute renal failure: Not reported  %patients with acute respiratory failure: Not reported  Mechanical ventilation at NOAF onset: Not reported  Serum potassium: Not reported  Definition of NOAF: Not reported | Switching therapy from landiolol to a bisoprolol patch  Switching occurred where a continuous landiolol infusion was used for AF-related tachycardia, and its administration duration reached 6 days, or where long-term therapy was expected before Day 6.  A 4mg/24h bisoprolol patch was attached and the landiolol infusion was stopped after 6h.  Median landiolol administration time before bisoprolol patch use: 88.1h. Median landiolol dosage on bisoprolol patch use: 3.1μg/kg/min. Median noradrenaline dosage on bisoprolol patch use: 0.20μg/kg/min.  Line of NOAF treatment:  Second line |
| Authors  Slavik et al. (2003)(18) (conference abstract)  Setting  General ICU  Country  Canada  Sample size  Not reported  %NOAF patients  6.6% patients developed NOAF | Primary diagnosis: Not reported  Mean age: Not reported  %Male: Not reported  Severity of illness: Not reported  %patients on vasopressors: Not reported  %patients with CVD: Not reported  %patients with acute renal failure: Not reported  %patients with acute respiratory failure: Not reported  %Mechanical ventilation at NOAF onset: Not reported  Serum potassium: Not reported  Definition of NOAF: Not reported | For NOAF rate control and conversion: IV amiodarone used in 74.5% episodes  For stroke prophylaxis: IV heparin used in 36.4% episodes  Line of NOAF treatment:  Not specified |
| Authors  Sleeswijk et al. 2008(19)  Setting  12-bed tertiary ICU  Country  The Netherlands  Sample size  n=29  %NOAF patients  100% | Primary diagnosis  Medical patients: n=11 (magnesium responders), n=9 (magnesium non responders)  Surgery patients : n=5 (magnesium responders), n=4 (magnesium non responders)  Primary diagnosis not reported  Mean age  64±16 years (magnesium responders), 69±17 years (magnesium non-responders)  % Male  n=14 (48%): n=7 (magnesium responders), n=7 (magnesium non-responders)  Severity of illness  Mean Apache II score reported: 19 ± 7 (all patients); 18 ± 7 (magnesium responders), 21 ±7 (magnesium non-responders)  % patients on vasopressors  n=17 (59%) (all patients); n=8 (50%) (magnesium responders), n=9 (69%) (magnesium non responders)  % patients with CVD  n=14 (48%) (all patients); n=5 (31%) (magnesium responders), n=9 (69%) (magnesium non responders)  % patients with acute renal failure: Not reported  % patients with acute respiratory failure: Not reported  Mechanical ventilation at NOAF onset: Not reported  Serum potassium: Not reported  Definition of NOAF: “New-onset AF was defined as newly developed AF during the ICU stay in patients without a previous history of atrial tachyarrhythmias and antiarrhythmic drug use. Diagnosis was confirmed by a 12-lead electrocardiogram (ECG).” | Magnesium infusion.  Patients received MgSO_4_ bolus (0.037g/kg) followed by continuous infusion (0.025g/kg/h).  The infusion rate was reduced to half when plasma [Mg^2+^] was >2.0 mmol/L. The infusion was stopped and stopped when plasma [Mg^2+^] was >2.0 mmol/L.  Where sinus rhythm was achieved, the infusion was stopped at the discretion of the treating clinician.  Line of NOAF treatment:  First line  Where no rhythm or rate control (<110bpm) was achieved after 1 hour of starting the MgSO_4_ infusion, an infusion of amiodarone (loading dose of 300mg followed by an infusion of 1200mg/24 hours) was started. Where sinus rhythm was achieved, the amiodarone infusion was stopped at the discretion of the treating clinician.  Line of NOAF treatment  Second line |

Supplementary table 8 - Results of prospective single group studies

| Authors | Results | Adverse effects | Recommendations/ barriers for the future research |
| --- | --- | --- | --- |
| Delle Karth et al. (2005)(14) | The authors reported that 82.4% patients converted back to sinus rhythm. The median (range) ICU length of stay was reported as 35 (13–44) days.  The mean time to termination of the arrhythmia was 17.7 ± 12.5 minutes (range 4 to 45 min) after start of the first infusion. The total administered dose to those who were successfully converted with ibutilide ranged from 0.5 to 2 mg (mean 1.20 ± .47 mg). | Sustained polymorphic ventricular tachycardia reported in one patient and this required emergency direct current cardioversion. Repetitive ventricular salvos was reported in two patients and ibutilide therapy had to be discontinued. Increased ventricular premature complexes were reported in 12 patients but ibutilide infusion did not have to be stopped. One patient experienced a ventricular pause of 3 seconds before conversion to sinus rhythm. | Not reported |
| Hennersdorf et al. (2002)(15) | Conversion to sinus rhythm was achieved in 71% (n=5) patients with recent-onset atrial fibrillation. The remaining patients were converted successfully by external electrical cardioversion. | Torsade de pointes (n=3/26 (11.5%)) | Not reported |
| Mayr et al. (2003)(16) | 13 patients (35%; 95% CI, 20–53%) primarily responded to DCC with restoration of SR, of which eight patients remained in SR (24%; CI, 12–41%) at 1 hr. At 24 and 48 hrs, six (16%; CI, 6–32%) and five (13.5%; CI, 5–29%) patients remained in SR, respectively.  Eight patients converted back to SR in response to the first DCC shock and four patients to the second DCC shock. One out of 22 patients returned to sinus rhythm after three DCC shocks, whereas no patient responded to a fourth DCC shock. | Not reported | “The optimal therapeutic regimen for effective and rapid termination of new-onset SVT in surgical ICU patients still remains to be established”. |
| Nakamura et al. (2016)(17) | Survival was achieved in 81.3 % of the patients in whom switching therapy was administered. The authors reported that three patients (18.75%) died of primary diseases after >3 days from switching therapy. In all patients who survived, bisoprolol patch therapy was continued on the ward. | The authors reported that there were no obvious adverse events in any patient and switching therapy was successfully completed in all the patients. | “It would be worth to conduct the further study investigating the efficacy of this switching therapy in the future.” |
| Slavik et al. (2003)(18) (abstract) | Authors reported that optimal rate control and conversion was achieved in 47% of NOAF episodes.  Authors reported that optimal stroke prophylaxis was achieved in 91% of NOAF episodes.  Note: Appropriateness of therapy assessed as optimal, appropriate and inappropriate; however, definitions not reported in the abstract | IV amiodarone: hypotension (35.6%)  IV heparin: major bleeding (5%) | Not reported |
| Sleeswijk et al. (2008)(19) | Seven patients converted to sinus rhythm within 1 hour after the start of the MgSO4 infusion, whereas 9 patients had a decrease in ventricular rate <110 beats/min. All patients achieved cardioversion after the start of the MgSO4 infusion without any additional therapy. Mean (SD) and median (range) time until conversion in the magnesium responders was 7 (± 11) hours and 2 (1-45) hours, respectively. Addition of amiodarone after 1 hour of the MgSO4 infusion was required for 13 patients (magnesium non-responders). Of these 13 patients, 11 achieved cardioversion within 24 hours. Mean (SD) and median (range) conversion time in magnesium non responders was 13 (± 21) hours and 4 (2-78) hours, respectively. The total mean (SD) and median (range) conversion time was 9.3 (± 16.3) hours and 3 (1-78) hours, respectively. The 24-hour conversion rate in the whole study group was 90%. Magnesium responders and non-responders were analysed separately. | Authors reported that no serious events that would cause the discontinuation of the treatment were observed during the study. | “A randomized controlled trial is needed to investigate whether this strategy is superior to other treatment regimes.” |

## Retrospective non-comparative studies

Supplementary table 9 - Methods and characteristics of retrospective single group studies

| Study details | Population characteristics | Intervention |
| --- | --- | --- |
| Authors  Burris et al. (2010)(20)  Setting  Surgical ICU  Country  USA  Sample size  n=30  Note: data extracted only for patients with atrial arrythmias (total study n=120. This includes controls that did not develop arrythmias)  %NOAF patients  Not reported | Primary diagnosis  General surgery 60%, vascular surgery 33%, orthopaedics 3.3%, neurosurgery 3.3%  Mean age: 66.2 ± 7.3 years  %Male: Not reported  Severity of illness: Not reported  %patients on vasopressors: n=14 (46.6%) (intraoperative)  %patients with CVD: CAD 40%, CHF 16.7%  %patients with acute renal failure: Not reported  %patients with acute respiratory failure: Not reported  Mechanical ventilation at NOAF onset: Not reported  Serum potassium: 3.9 ± .66 mmol/L (preoperative)  Definition of NOAF: Not reported | Amiodarone (63%)  Metoprolol (26%)  Esmolol (13.3%)  Diltiazem (6.7%)  Digoxin (3.3%)  Multiple drug regimens (10%)  Electrical cardioversion (only in combination with pharmacological treatment) n=4 (13.3%)  Line of NOAF treatment  Not specified for each treatment |
| Authors  Kanji et al. (2012)(21)  Setting  Three academic mixed medical/ surgical ICUs  Country  Canada  Sample size  n=139  %NOAF patients  100% | Primary diagnosis  Admission diagnosis reported: sepsis 18%, respiratory failure/ pneumonia 19%, cardiogenic shock/cardiac arrest 4%, cerebrovascular accident 1%, rapid AF 4%, postoperative care 48%, other 6%  Mean age: 71.6 ± 12.5 years  %Male: n=83 (60%)  Severity of illness  Mean APACHE II score reported: 22.6 ± 9.0  %patients on vasopressors: Not reported  %patients with CVD: Coronary artery disease 29%, valvular heart disease 1%, Congestive heart failure 6%, cardiomyopathy (dilated, hypertrophic) 2%  %patients with acute renal failure: Not reported  %patients with acute respiratory failure: n=27 (19%)  Mechanical ventilation at NOAF onset: Not reported  Serum potassium: <3.5 mmol/L, n=15 (11%)  Definition of NOAF: “Definition for NOAF cases reported as “defined as those with no previous documented history of any atrial arrhythmia documented in the physical or electronic medical record” | Rhythm control attempted (n=105) by administering intravenous amiodarone.  Rate control attempted (n=28) by administering β-blockers, calcium-channel blockers, or digoxin, alone or in combination.  Line of NOAF treatment  Not specified |
| Authors  Kyo et al. (2019)(22)  Setting  Two mixed ICUs (emergency and medicosurgical ICU)  Country  Japan  Sample size  n=85  %NOAF patients  100% | Primary diagnosis   \|  \| All patients  (n=85) \| Successful ECV (n=41) \| Unsuccessful ECV (n=44) \| \| --- \| --- \| --- \| --- \| \| Cardiovascular, n (%) \| 33 (38) \| 14 (34) \| 19 (43) \| \| Pulmonary, n (%) \| 26 (31) \| 15 (37) \| 11 (25) \| \| Gastrointestinal, n (%) \| 10 (12) \| 4 (10) \| 6 (14) \| \| Neurology, n (%) \| 4 (5) \| 1 (2) \| 3 (7) \| \| Trauma, n (%) \| 3 (4) \| 3 (7) \| - \| \| Skin and soft tissue, n (%) \| 5 (6) \| 2 (5) \| 3 (7) \| \| Other, n (%) \| 4 (5) \| 2 (5) \| 2 (5) \|   Mean age  median (range): 71 (64–78) (all patients), 71 (64–79) (successful ECV), 71 (62–77) (unsuccessful ECV)  %Male  n=58 (68%) (all patients), n=30 (73%) (successful ECV), n=28 (64%) (unsuccessful ECV)  Severity of illness  APACHE II score at ICU admission, median (range): 26 (17–34) (all patients), 26 (18–35) (successful ECV), 26 (16–31) (unsuccessful ECV)  SOFA score at the onset of AF, median (range): 8 (5-11) (all patients), 8 (5-12) (successful ECV), 8 (5-9) (unsuccessful ECV)  %patients on vasopressors   \|  \| All patients  (n=85) \| Successful ECV (n=41) \| Unsuccessful ECV (n=44) \| \| --- \| --- \| --- \| --- \| \| Norepinephrine \| 24 (28) \| 12 (29) \| 12 (27) \| \| Dopamine \| 21 (25) \| 10 (24) \| 11 (25) \| \| Dobutamine \| 25 (29) \| 14 (34) \| 11 (25) \|   %patients with CVD: Medical history not reported  %patients with acute renal failure: Not reported  %patients with acute respiratory failure: Not reported  Mechanical ventilation at NOAF onset  Not specified whether at the time of onset  n=71 (84%) (all patients), n=37 (90%) (successful ECV), n=34 (77%) (unsuccessful ECV)  Serum potassium (mmol/L)  Median, range: 4.0 (3.7–4.6) (all patients), 4.2 (3.9–4.8) (successful ECV), 3.9 (3.6–4.3) (unsuccessful ECV)  Definition of NOAF: “New-onset AF was defined as the first AF rhythm on ECG occurring during an ICU stay” | Electrical cardioversion (ECV)  Median of one (IQR, 1–2) shock per ECV session.  Line of NOAF treatment  Not specified |
| Authors  Liu et al. (2016)(23)  Setting  Medical ICU  Country  Taiwan  Sample size  n=240  %NOAF patients  100% | Primary diagnosis  Sepsis  Infection site   \|  \| NOAF to sinus rhythm (SR) (n=165) \| NOAF to atrial fibrillation (AF) (n=75) \| \| --- \| --- \| --- \| \| Respiratory tract, n (%) \| 112 (67.9) \| 48 (64) \| \| Urinary tract, n (%) \| 35 (21.2) \| 14 (18.7) \| \| Gastrointestinal, n (%) \| 9 (5.5) \| 5 (6.7) \| \| Other, n (%) \| 9 (5.5) \| 8 (10.7) \|   Mean age: 77.8 ± 10.3 (NOAF to SR), 76.2 ± 11.0 (NOAF to AF)  %Male: n= 90 (54.5%) (NOAF to SR), n= 46 (61.3%) (NOAF to AF)  Severity of illness  Mean SOFA score reported: 7.6 ± 3.0 (NOAF to SR), 9.3 ± 3.2 (NOAF to AF)  Mean APACHE II score: 22.8 ± 5.8 (NOAF to SR), 24.6 ± 6.1 (NOAF to AF)  %patients on vasopressors   \|  \| NOAF to SR (n=165) \| NOAF to AF  (n=75) \| \| --- \| --- \| --- \| \| Dopamine, n (%) \| 64 (38.8) \| 49 (65.3) \| \| Norepinephrine, n (%) \| 58 (35.2) \| 48 (64) \|   %patients with CVD   \|  \| NOAF to SR (n=165) \| NOAF to AF  (n=75) \| \| --- \| --- \| --- \| \| Heart failure, n (%) \| 35 (21.2) \| 15 (20) \| \| Coronary artery disease, n (%) \| 70 (42.4) \| 37 (49.3) \|   %patients with acute renal failure: n=65 (39.4%) (NOAF to SR), n=39 (52.0%) (NOAF to AF)  %patients with acute respiratory failure: n=150 (90.9%) (NOAF to SR), n=71 (94.7%) (NOAF to AF)  Mechanical ventilation at NOAF onset: n=143 (86.7%) (NOAF to SR), n=69 (92.0%) (NOAF to AF)  Serum potassium (mmol/L): 4.2 ± 1.0 (NOAF to AF), 4.1 ± 0.9 (NOAF to SR)  Definition of NOAF  “The absence of P waves and irregular ventricular activity lasting for more than 30 seconds”  NOAF to AF defined as “persistent or recurrent AF 7 days after the onset of NOAF” | Amiodarone (n=80 (33.3%))  Beta-blockers (n=88 (36.7%))  Non-dihydropyridine calcium channel blockers (n=66 (27.5%))  Digoxin glycosides (n=27 (11.3%))  Electrical cardioversion (n=8 (3.3%))  Line of NOAF treatment  Not specified for each treatment |
| Authors  Mayr et al. (2004)(24)  Setting  12-bed general and surgical ICU in a university teaching hospital  Country  Austria  Sample size  N=131  %NOAF patients  93% | Primary diagnosis  Type of surgery   \|  \| All patients  (n=131) \| Responders  (n=98) \| Non-responders (n=33) \| \| --- \| --- \| --- \| --- \| \| Cardiac, n (%) \| 61 (46.56) \| 46 (46.9) \| 15 (45.45) \| \| General, n (%) \| 53 (40.45) \| 41 (41.8) \| 12 (36.36) \| \| Vascular, n (%) \| 7 (5.3) \| 5 (5.1) \| 2 (6.2) \| \| Trauma, n (%) \| 5 (3.8) \| 2 (2) \| 3 (9.1) \| \| Orthopaedic, n (%) \| 5 (3.8) \| 4 (4.1) \| 1 (3) \|   Sepsis: 23.9% (all patients), 24% (responders), 23.6 % (non-responders)  Mean age (years): 68 ± 12 (all patients), 68 ± 12 (responders), 67 ± 14 (non-responders)  %Male: n=82 (62.6%) (all patients), n= 58 (59.2%) (responders), n= 24 (72.7%) (non-responders)  Severity of illness  Mean MODS score reported: 7.5 ± 3.4 (all patients), 7.4 ± 3.4 (responders), 7.9 ± 3.5 (non-responders)  %patients on vasopressors: Not reported  %patients with CVD: Not reported  %patients with acute renal failure: Not reported  %patients with acute respiratory failure: Not reported  Mechanical ventilation at NOAF onset: Not reported  Serum potassium: Not reported  Definition of NOAF: “New-onset supraventricular tachyarrhythmias were defined as “narrow-complex non-sinus tachyarrhythmias with heart rates ≥100 bpm lasting for longer than 30 minutes”. | Amiodarone infusion.  Amiodarone was infused via central venous catheter at 90 mg/h for maximum of 12 hours, followed by a weaning regimen (initially 40– 60 mg/h for maximum of 3 days, then 20 mg/h for another 5–7 days.  Amiodarone was continued orally (200 mg TDS) in some patients.  The amiodarone infusion was stopped where the heart rate dropped below 60 bpm.  Line of NOAF treatment:  First line |
| Authors  Mitric et al. (2016)(25)  Setting  Medical-surgical trauma ICU  Country  Australia  Sample size  n=177  n=86 (no recurrence of AF)  n=91 (recurrence of AF)  %NOAF patients  100% | Primary diagnosis: Not reported  Mean age (years)  Median (range) age: 69 (60–75) (all patients), 65 (57–75) (no recurrence of AF), 71 (61–76) (recurrence of AF)  %Male: n=113 (64%) (all patients), n=53 (61%) (no recurrence of AF), n=60 (66%) (recurrence of AF)  Severity of illness  Median (range) APACHE II score reported: 22 (17–28) (all patients), 21 (17–26) (no recurrence of AF), 23 (17–29) (recurrence of AF)  Median (range) SAPS II score reported: 41 (31–53) (all patients), 39 (30–49) (no recurrence of AF), 44 (31–58) (recurrence of AF)  Median (range) Charlson Score reported: 2 (1–4) (all patients), 2 (1–4) (no recurrence of AF), 3 (2–5) (recurrence of AF)  %patients on vasopressors: Not reported  %patients with CVD   \|  \| All patients  (n=177) \| No recurrent AF (n=86) \| Recurrent AF  (n=91) \| \| --- \| --- \| --- \| --- \| \| Myocardial infarction, n (%) \| 43 (24) \| 17 (20) \| 26 (29) \| \| Congestive cardiac failure, n (%) \| 22 (12) \| 6 (7) \| 16 (18) \| \| Ischaemic heart disease, n (%) \| 58 (33) \| 23 (27) \| 35 (38) \| \| Rheumatic heart disease, n (%) \| 2 (1) \| 2 (2) \| 0 (0) \| \| Mitral valve disease, n (%) \| 9 (5) \| 7 (8) \| 2 (2) \|   %patients with acute renal failure: Not reported  %patients with acute respiratory failure: Not reported  Mechanical ventilation at NOAF onset: Not reported  Serum potassium (mmol/L)  Reported as median (IQR) only for the recurrent AF group   \|  \| On the day AF initially reverted (n=81)* \| On day AF recurred  (n=73)* \| \| --- \| --- \| --- \| \| K min \| 4.2 (3.8–4.5) \| 4.2 (4.0–4.6) \| \| K max \| 4.3 (4.1–4.7) \| 4.4 (4.1–4.7) \|   *Differing patient numbers due to missing data  Definition of NOAF: Defined as “a rhythm on the electrocardiogram (ECG) with replacement of P waves with rapid oscillations or fibrillatory waves that vary in size, shape and timing, associated with an irregular, frequently rapid, ventricular response when atrioventricular conduction is intact” | Amiodarone  Information was gathered about nature of administration (bolus, infusion, or both, and presence of any delays to administration.   \|  \| All patients  (n=177) \| No AF recurrence (n=86) \| AF recurrence (n=91) \| \| --- \| --- \| --- \| --- \| \| Amiodarone dosing, n (%)  *Bolus only* \| 23 (13) \| 3 (3) \| 20 (23) \| \| *Infusion only* \| 62 (35) \| 43 (50) \| 19 (25) \| \| *Bolus and infusion* \| 92 (52) \| 40 (47) \| 52 (57) \| \| Delay to infusion after bolus, hours (median (IQR)) \| 2 (1–4)  (n = 74) \| 2 (1–3)  (n = 29) \| 2 (1–6)  (n = 45) \| \| Total dose, mg (median (IQR)) \| 905  (488–1651) \| 702  (300–1117) \| 1366  (752–2711) \|   Line of NOAF treatment  First line |

Supplementary table 10 - Results of retrospective single group studies

| Authors | Results | Adverse effects | Recommendations/ barriers for the future research |
| --- | --- | --- | --- |
| Burris et al. (2010)(20) | Authors reported that 33% of patients treated with amiodarone achieved successful conversion to sinus rhythm.  Ten percent of all patients did not convert to sinus rhythm, and 6.7% of those who converted later reverted to arrhythmia. | Not reported | “Randomized prospective studies are required to determine the success of alternative treatments and should provide the evidence needed to streamline management of this problem.” |
| Kanji et al. (2012)(21) | Rhythm control alone was attempted in 105 patients (103 patients were administered intravenous amiodarone and 2 patients were administered sotalol). Successful rhythm conversion was achieved in 90 (87%) patients at some point while receiving amiodarone. 38 (42%) of 90 patients reverted back to AF during their ICU stay after maintaining normal sinus rhythm for at least 24 hours after cardioversion. Authors reported that of the patients with successful rhythm conversion (n=90), 51 (57%) converted within 6 hours, and 66 (73%) converted within 24 hours. Of the 74 patients in this group who were discharged from ICU, 13 (18%) left the ICU in AF. Two patients treated with sotalol converted to sinus rhythm, but 1 patient reverted back to AF after being in sinus rhythm for 24 hours. Both patients (who received sotalol) were discharged from the ICU in normal sinus rhythm.  Twenty-eight (20%) patients were treated with rate-controlling agents alone (β-blockers, calcium-channel blockers, or digoxin, alone or in combination). Twenty-one (75%) patients converted to sinus rhythm while receiving rate-control therapy alone, and 5 (19%) of 27 ICU survivors were discharged from the ICU in AF. | Not reported | In the general adult critically ill population, more research is required to determine; 1) Whether attempting rhythm control is more effective at restoring sinus rhythm than attempting rate control alone; 2) whether attempting rhythm control improves clinical outcomes; and 3) what is the optimal anticoagulation strategy in patients who develop NOAF. |
| Kyo et al. (2019)(22) | Electrical cardioversion was successful in 41 (48%) patients. From these, eleven (13%) patients maintained sinus rhythm until ICU discharge and 30 (35%) patients had recurrent AF. Among the 44 (52%) patients with unsuccessful ECV, 7 (8%) did not convert back to sinus rhythm until after ICU discharge, while 37 (44%) converted to sinus rhythm during their ICU stay.  Authors reported no difference in median length of ICU stay (days) between the patients who had successful ECV and those who had unsuccessful ECV (16 (11-17) versus 15 (7-23), respectively).  No difference in ICU deaths was observed between groups (n=16 (39%) in successful ECV group versus 14 (32%) in unsuccessful ECV group.  Similarly, no difference in median length of hospital stay (days) was found between the patient groups (28 (16-62) in successful ECV group versus 31 (19-60) in unsuccessful ECV group).  Authors also did not find any difference in hospital death between the patients with successful ECV and unsuccessful ECV (21 (51%) versus 17 (39%), respectively). | Not reported | “Further studies are needed to investigate the potential factors associated with the maintenance of SR to establish a better understanding of new-onset AF in critically ill patients.” |
| Liu et al. (2016)(23) | Fifty-two from 80 patients (65%) who received amiodarone converted to sinus rhythm.  Sixty-seven from 88 patients (76.1%) who were treated with beta-blockers converted back to sinus rhythm.  Forty-seven from 66 patients (71.21%) who were treated with Non-DHP CCBs converted to sinus rhythm.  Fifteen from 27 patients (55.55%) treated with digoxin glycosides converted back to sinus rhythm.  Fifty percent of patients who were treated with electrical cardioversion converted back to sinus rhythm. | Not reported | “A larger, prospective comparative study is needed to elucidate the clinical implications between a rate control and a rhythm control strategy in patients with sepsis and NOAF”. |
| Mayr et al. (2004)(24) | Sinus rhythm was achieved in 54.2% of patients within the first 12 hours, in 64% within 24 hours and in 74.8% within 48 hours. Heart rate decreased significantly in all patients (–37%) during the observation period, but the decrease was more pronounced in responders than in non-responders.  The authors reported no differences in the length of SICU stay between responders and non-responders (13 ± 10 days versus 14 ± 11 days, respectively. It was reported that there was a trend of higher SICU mortality in non-responders (39.4%) than in responders (24.5%) (p=0.1; 28.2% in all patients). | Increases in serum concentrations of creatinine and bilirubin observed. | Not reported |
| Mitric et al. (2016)(25) | Eighty-six (49 %) patients were successfully treated with amiodarone, without recurrence of AF until discharge from ICU. The recurrence of AF occurred in 91 patients (51 %) at least once during the ICU stay, after initial successful conversion to normal sinus rhythm.  Addition of amiodarone infusion, and continuing the infusion to ICU discharge were associated with less recurrent arrhythmia.  The median ICU length of stay was reported as 7 (4-13) days in all patients; 6 (3-12) days in patients who had no recurrence of AF and 8 (4-16) days in patients who had recurrence of AF.  The median hospital length of stay was reported as 25 (13–58), 21 (12–46) and 31 (18–70) for all patients, patients who had no recurrence of AF and patients who had recurrence of AF, respectively.  In total, 23 (13%) of all patients died on an ICU, 10 (12%) of patients who had no recurrence of AF and 13 (14%) of patients who had recurrence of AF.  Forty-seven (17%) of all patients died in the hospital, 22 (26%) of patients who had no recurrent AF and 25 (27%) of patients who had recurrent AF. | Not reported | “A clear dosing guide is not available and further research is required to elicit the best dosing strategy.” |

# Critical appraisal

## Risk of bias assessment for randomized trials

Revised Cochrane risk-of-bias tool for randomized trials (RoB 2)

TEMPLATE FOR COMPLETION

Edited by Julian PT Higgins, Jelena Savović, Matthew J Page, Jonathan AC Sterne
on behalf of the RoB2 Development Group

**Version of 22 August 2019**

The development of the RoB 2 tool was supported by the MRC Network of Hubs for Trials Methodology Research (MR/L004933/2- N61), with the support of the host MRC ConDuCT-II Hub (Collaboration and innovation for Difficult and Complex randomised controlled Trials In Invasive procedures - MR/K025643/1), by MRC research grant MR/M025209/1, and by a grant from The Cochrane Collaboration.


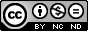


This work is licensed under a [Creative Commons Attribution-NonCommercial-NoDerivatives 4.0 International License](http://creativecommons.org/licenses/by-nc-nd/4.0/).

| **Study details**   \| **Reference** \| Delle Karth G, Geppert A, Neunteufl T, Priglinger U, Haumer M, Gschwandtner M, et al. Amiodarone versus diltiazem for rate control in critically ill patients with atrial tachyarrhythmias. Crit Care Med 2001;29:1149-53. <http://dx.doi.org/10.1097/00003246-200106000-00011> \| \| --- \| --- \|   **Study design**   \| X \| Individually-randomized parallel-group trial \| \| --- \| --- \| \| □ \| Cluster-randomized parallel-group trial \| \| □ \| Individually randomized cross-over (or other matched) trial \|   **For the purposes of this assessment, the interventions being compared are defined as**   \| Experimental: \| Amiodarone \| Comparator: \| Diltiazem \| \| --- \| --- \| --- \| --- \|  \| **Specify which outcome is being assessed for risk of bias** \| Rate control \| \| --- \| --- \|  \| **Specify the numerical result being assessed.** In case of multiple alternative analyses being presented, specify the numeric result (e.g. RR = 1.52 (95% CI 0.83 to 2.77) and/or a reference (e.g. to a table, figure or paragraph) that uniquely defines the result being assessed. \|  \| \| --- \| --- \|   **Is the review team’s aim for this result…?**   \| X \| to assess the effect of *assignment to intervention* (the ‘intention-to-treat’ effect) \| \| --- \| --- \| \| □ \| to assess the effect of *adhering to intervention* (the ‘per-protocol’ effect) \|   **If the aim is to assess the effect of *adhering to intervention***, select the deviations from intended intervention that should be addressed (at least one must be checked):  □ occurrence of non-protocol interventions  □ failures in implementing the intervention that could have affected the outcome  □ non-adherence to their assigned intervention by trial participants  **Which of the following sources were obtained to help inform the risk-of-bias assessment? (tick as many as apply)**  X Journal article(s) with results of the trial  □ Trial protocol  □ Statistical analysis plan (SAP)  □ Non-commercial trial registry record (e.g. ClinicalTrials.gov record)  □ Company-owned trial registry record (e.g. GSK Clinical Study Register record)  □ “Grey literature” (e.g. unpublished thesis)  □ Conference abstract(s) about the trial  □ Regulatory document (e.g. Clinical Study Report, Drug Approval Package)  □ Research ethics application  □ Grant database summary (e.g. NIH RePORTER or Research Councils UK Gateway to Research)  □ Personal communication with trialist  □ Personal communication with the sponsor |
| --- | --- | --- | --- | --- | --- | --- | --- | --- | --- | --- | --- | --- | --- | --- | --- | --- | --- | --- | --- | --- |

Risk of bias assessment

Responses underlined in green are potential markers for low risk of bias, and responses in red are potential markers for a risk of bias. Where questions relate only to sign posts to other questions, no formatting is used.

**Domain 1: Risk of bias arising from the randomization process**

| **Signalling questions** | **Comments** | **Response options** |
| --- | --- | --- |
| **1.1 Was the allocation sequence random?** | Patients were "randomly assigned" to treatments | NI |
| **1.2 Was the allocation sequence concealed until participants were enrolled and assigned to interventions?** |  | NI |
| **1.3 Did baseline differences between intervention groups suggest a problem with the randomization process?** | Important baseline differences in sex and age (though could also be due to the play of chance). | PY |
| **Risk-of-bias judgement** |  | High |
| Optional: What is the predicted direction of bias arising from the randomization process? |  | NA / Favours experimental / Favours comparator / Towards null /Away from null / Unpredictable |

Domain 2: Risk of bias due to deviations from the intended interventions (*effect of assignment to intervention*)

| **Signalling questions** | **Comments** | **Response options** |
| --- | --- | --- |
| **2.1. Were participants aware of their assigned intervention during the trial?** | Participants in intensive care. No blinding of interventions given. | PN |
| **2.2. Were carers and people delivering the interventions aware of participants' assigned intervention during the trial?** |  | Y |
| **2.3. If Y/PY/NI to 2.1 or 2.2: Were there deviations from the intended intervention that arose because of the trial context?** |  | NI |
| **2.4 If Y/PY to 2.3: Were these deviations likely to have affected the outcome?** |  | NA |
| **2.5. If Y/PY/NI to 2.4: Were these deviations from intended intervention balanced between groups?** |  | NA |
| **2.6 Was an appropriate analysis used to estimate the effect of assignment to intervention?** | Full ITT analysis | Y |
| **2.7 If N/PN/NI to 2.6: Was there potential for a substantial impact (on the result) of the failure to analyse participants in the group to which they were randomized?** |  | NA |
| **Risk-of-bias judgement** |  | Some concerns |
| Optional: What is the predicted direction of bias due to deviations from intended interventions? |  | NA / Favours experimental / Favours comparator / Towards null /Away from null / Unpredictable |

Domain 2: Risk of bias due to deviations from the intended interventions (*effect of adhering to intervention*)

| **Signalling questions** | **Comments** | **Response options** |
| --- | --- | --- |
| **2.1. Were participants aware of their assigned intervention during the trial?** |  | Y / PY / PN / N / NI |
| **2.2. Were carers and people delivering the interventions aware of participants' assigned intervention during the trial?** |  | Y / PY / PN / N / NI |
| **2.3. [If applicable:] If Y/PY/NI to 2.1 or 2.2: Were important non-protocol interventions balanced across intervention groups?** |  | NA / Y / PY / PN / N / NI |
| **2.4. [If applicable:] Were there failures in implementing the intervention that could have affected the outcome?** |  | NA / Y / PY / PN / N / NI |
| **2.5. [If applicable:] Was there non-adherence to the assigned intervention regimen that could have affected participants’ outcomes?** |  | NA / Y / PY / PN / N / NI |
| **2.6. If N/PN/NI to 2.3, or Y/PY/NI to 2.4 or 2.5: Was an appropriate analysis used to estimate the effect of adhering to the intervention?** |  | NA / Y / PY / PN / N / NI |
| **Risk-of-bias judgement** |  | Low / High / Some concerns |
| Optional: What is the predicted direction of bias due to deviations from intended interventions? |  | NA / Favours experimental / Favours comparator / Towards null /Away from null / Unpredictable |

Domain 3: Missing outcome data

| **Signalling questions** | **Comments** | **Response options** |
| --- | --- | --- |
| **3.1 Were data for this outcome available for all, or nearly all, participants randomized?** |  | PY |
| **3.2 If N/PN/NI to 3.1: Is there evidence that the result was not biased by missing outcome data?** |  | NA |
| **3.3 If N/PN to 3.2: Could missingness in the outcome depend on its true value?** |  | NA |
| **3.4 If Y/PY/NI to 3.3: Is it likely that missingness in the outcome depended on its true value?** |  | NA |
| **Risk-of-bias judgement** |  | Low |
| Optional: What is the predicted direction of bias due to missing outcome data? |  | NA / Favours experimental / Favours comparator / Towards null /Away from null / Unpredictable |

Domain 4: Risk of bias in measurement of the outcome

| **Signalling questions** | **Comments** | **Response options** |
| --- | --- | --- |
| **4.1 Was the method of measuring the outcome inappropriate?** |  | N |
| **4.2 Could measurement or ascertainment of the outcome have differed between intervention groups?** |  | PN |
| **4.3 If N/PN/NI to 4.1 and 4.2: Were outcome assessors aware of the intervention received by study participants?** |  | PY |
| **4.4 If Y/PY/NI to 4.3: Could assessment of the outcome have been influenced by knowledge of intervention received?** | Sustained rate reduction of 30% is an objective outcome | PN |
| **4.5 If Y/PY/NI to 4.4:** **Is it likely that assessment of the outcome was influenced by knowledge of intervention received?** |  | NA |
| **Risk-of-bias judgement** |  | Low |
| Optional: What is the predicted direction of bias in measurement of the outcome? |  | NA / Favours experimental / Favours comparator / Towards null /Away from null / Unpredictable |

Domain 5: Risk of bias in selection of the reported result

| **Signalling questions** | **Comments** | **Response options** |
| --- | --- | --- |
| **5.1 Were the data that produced this result analysed in accordance with a pre-specified analysis plan that was finalized before unblinded outcome data were available for analysis?** |  | NI |
| **Is the numerical result being assessed likely to have been selected, on the basis of the results, from...** |  |  |
| **5.2. ... multiple eligible outcome measurements (e.g. scales, definitions, time points) within the outcome domain?** |  | NI |
| **5.3 ... multiple eligible analyses of the data?** |  | NI |
| **Risk-of-bias judgement** |  | Some concerns |
| Optional: What is the predicted direction of bias due to selection of the reported result? |  | NA / Favours experimental / Favours comparator / Towards null /Away from null / Unpredictable |

Overall risk of bias

| **Risk-of-bias judgement** |  | High |
| --- | --- | --- |
| Optional: What is the overall predicted direction of bias for this outcome? |  | NA / Favours experimental / Favours comparator / Towards null /Away from null / Unpredictable |

| **Study details**   \| **Reference** \| Balser JR, Martinez EA, Winters BD, Perdue PW, Clarke AW, Huang W, et al. Beta-adrenergic blockade accelerates conversion of postoperative supraventricular tachyarrhythmias. Anesthesiology 1998;89:1052-9. <http://dx.doi.org/10.1097/00000542-199811000-00004> \| \| --- \| --- \|   **Study design**   \| X \| Individually-randomized parallel-group trial \| \| --- \| --- \| \| □ \| Cluster-randomized parallel-group trial \| \| □ \| Individually randomized cross-over (or other matched) trial \|   **For the purposes of this assessment, the interventions being compared are defined as**   \| Experimental: \| Diltiazem \| Comparator: \| Esmolol \| \| --- \| --- \| --- \| --- \|  \| **Specify which outcome is being assessed for risk of bias** \| Rhythm control \| \| --- \| --- \|  \| **Specify the numerical result being assessed.** In case of multiple alternative analyses being presented, specify the numeric result (e.g. RR = 1.52 (95% CI 0.83 to 2.77) and/or a reference (e.g. to a table, figure or paragraph) that uniquely defines the result being assessed. \| 2hr conversion subgroup of AFib patients \| \| --- \| --- \|   **Is the review team’s aim for this result…?**   \| X \| to assess the effect of *assignment to intervention* (the ‘intention-to-treat’ effect) \| \| --- \| --- \| \| □ \| to assess the effect of *adhering to intervention* (the ‘per-protocol’ effect) \|   **If the aim is to assess the effect of *adhering to intervention***, select the deviations from intended intervention that should be addressed (at least one must be checked):  □ occurrence of non-protocol interventions  □ failures in implementing the intervention that could have affected the outcome  □ non-adherence to their assigned intervention by trial participants  **Which of the following sources were obtained to help inform the risk-of-bias assessment? (tick as many as apply)**  X Journal article(s) with results of the trial  □ Trial protocol  □ Statistical analysis plan (SAP)  □ Non-commercial trial registry record (e.g. ClinicalTrials.gov record)  □ Company-owned trial registry record (e.g. GSK Clinical Study Register record)  □ “Grey literature” (e.g. unpublished thesis)  □ Conference abstract(s) about the trial  □ Regulatory document (e.g. Clinical Study Report, Drug Approval Package)  □ Research ethics application  □ Grant database summary (e.g. NIH RePORTER or Research Councils UK Gateway to Research)  □ Personal communication with trialist  □ Personal communication with the sponsor |
| --- | --- | --- | --- | --- | --- | --- | --- | --- | --- | --- | --- | --- | --- | --- | --- | --- | --- | --- | --- | --- |

Risk of bias assessment

Responses underlined in green are potential markers for low risk of bias, and responses in red are potential markers for a risk of bias. Where questions relate only to sign posts to other questions, no formatting is used.

**Domain 1: Risk of bias arising from the randomization process**

| **Signalling questions** | **Comments** | **Response options** |
| --- | --- | --- |
| **1.1 Was the allocation sequence random?** | Patients "were randomized" | NI |
| **1.2 Was the allocation sequence concealed until participants were enrolled and assigned to interventions?** |  | NI |
| **1.3 Did baseline differences between intervention groups suggest a problem with the randomization process?** |  | PN |
| **Risk-of-bias judgement** |  | Some concerns |
| Optional: What is the predicted direction of bias arising from the randomization process? |  | NA / Favours experimental / Favours comparator / Towards null /Away from null / Unpredictable |

Domain 2: Risk of bias due to deviations from the intended interventions (*effect of assignment to intervention*)

| **Signalling questions** | **Comments** | **Response options** |
| --- | --- | --- |
| **2.1. Were participants aware of their assigned intervention during the trial?** | Patients were in an ICU. Abstract says study was "unblinded" | PN |
| **2.2. Were carers and people delivering the interventions aware of participants' assigned intervention during the trial?** |  | Y |
| **2.3. If Y/PY/NI to 2.1 or 2.2: Were there deviations from the intended intervention that arose because of the trial context?** | Similar proportions of post-randomisation use of digoxin but no reporting on the use of "DC cardioversion implemented at the discretion of physician staff" | NI |
| **2.4 If Y/PY to 2.3: Were these deviations likely to have affected the outcome?** |  | NA |
| **2.5. If Y/PY/NI to 2.4: Were these deviations from intended intervention balanced between groups?** |  | NA |
| **2.6 Was an appropriate analysis used to estimate the effect of assignment to intervention?** |  | Y |
| **2.7 If N/PN/NI to 2.6: Was there potential for a substantial impact (on the result) of the failure to analyse participants in the group to which they were randomized?** |  | NA |
| **Risk-of-bias judgement** |  | Some concerns |
| Optional: What is the predicted direction of bias due to deviations from intended interventions? |  | NA / Favours experimental / Favours comparator / Towards null /Away from null / Unpredictable |

Domain 2: Risk of bias due to deviations from the intended interventions (*effect of adhering to intervention*)

| **Signalling questions** | **Comments** | **Response options** |
| --- | --- | --- |
| **2.1. Were participants aware of their assigned intervention during the trial?** |  | Y / PY / PN / N / NI |
| **2.2. Were carers and people delivering the interventions aware of participants' assigned intervention during the trial?** |  | Y / PY / PN / N / NI |
| **2.3. [If applicable:] If Y/PY/NI to 2.1 or 2.2: Were important non-protocol interventions balanced across intervention groups?** |  | NA / Y / PY / PN / N / NI |
| **2.4. [If applicable:] Were there failures in implementing the intervention that could have affected the outcome?** |  | NA / Y / PY / PN / N / NI |
| **2.5. [If applicable:] Was there non-adherence to the assigned intervention regimen that could have affected participants’ outcomes?** |  | NA / Y / PY / PN / N / NI |
| **2.6. If N/PN/NI to 2.3, or Y/PY/NI to 2.4 or 2.5: Was an appropriate analysis used to estimate the effect of adhering to the intervention?** |  | NA / Y / PY / PN / N / NI |
| **Risk-of-bias judgement** |  | Low / High / Some concerns |
| Optional: What is the predicted direction of bias due to deviations from intended interventions? |  | NA / Favours experimental / Favours comparator / Towards null /Away from null / Unpredictable |

Domain 3: Missing outcome data

| **Signalling questions** | **Comments** | **Response options** |
| --- | --- | --- |
| **3.1 Were data for this outcome available for all, or nearly all, participants randomized?** | Data for all 44 patients with atrial fibrillation were reported | Y |
| **3.2 If N/PN/NI to 3.1: Is there evidence that the result was not biased by missing outcome data?** |  | NA |
| **3.3 If N/PN to 3.2: Could missingness in the outcome depend on its true value?** |  | NA |
| **3.4 If Y/PY/NI to 3.3: Is it likely that missingness in the outcome depended on its true value?** |  | NA |
| **Risk-of-bias judgement** |  | Low |
| Optional: What is the predicted direction of bias due to missing outcome data? |  | NA / Favours experimental / Favours comparator / Towards null /Away from null / Unpredictable |

Domain 4: Risk of bias in measurement of the outcome

| **Signalling questions** | **Comments** | **Response options** |
| --- | --- | --- |
| **4.1 Was the method of measuring the outcome inappropriate?** |  | N |
| **4.2 Could measurement or ascertainment of the outcome have differed between intervention groups?** |  | N |
| **4.3 If N/PN/NI to 4.1 and 4.2: Were outcome assessors aware of the intervention received by study participants?** | Electrocardiograms and rhythm strips reviewed by a cardiologist who was blinded to treatment | N |
| **4.4 If Y/PY/NI to 4.3: Could assessment of the outcome have been influenced by knowledge of intervention received?** |  | NA |
| **4.5 If Y/PY/NI to 4.4: Is it likely that assessment of the outcome was influenced by knowledge of intervention received?** |  | NA |
| **Risk-of-bias judgement** |  | Low |
| Optional: What is the predicted direction of bias in measurement of the outcome? |  | NA / Favours experimental / Favours comparator / Towards null /Away from null / Unpredictable |

Domain 5: Risk of bias in selection of the reported result

| **Signalling questions** | **Comments** | **Response options** |
| --- | --- | --- |
| **5.1 Were the data that produced this result analysed in accordance with a pre-specified analysis plan that was finalized before unblinded outcome data were available for analysis?** |  | NI |
| **Is the numerical result being assessed likely to have been selected, on the basis of the results, from...** |  |  |
| **5.2. ... multiple eligible outcome measurements (e.g. scales, definitions, time points) within the outcome domain?** | Likely that there is only one way in which the outcome can be measured | PN |
| **5.3 ... multiple eligible analyses of the data?** | Outcome likely to be analysable in only one way | PN |
| **Risk-of-bias judgement** |  | Some concerns |
| Optional: What is the predicted direction of bias due to selection of the reported result? |  | NA / Favours experimental / Favours comparator / Towards null /Away from null / Unpredictable |

Overall risk of bias

| **Risk-of-bias judgement** |  | Some concerns |
| --- | --- | --- |
| Optional: What is the overall predicted direction of bias for this outcome? |  | NA / Favours experimental / Favours comparator / Towards null /Away from null / Unpredictable |


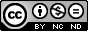


This work is licensed under a [Creative Commons Attribution-NonCommercial-NoDerivatives 4.0 International License](http://creativecommons.org/licenses/by-nc-nd/4.0/).

## Risk of bias assessment for large non-randomized studies

Supplementary table 11 - Risk of bias assessments for large non-randomised studies

| Study* | Domain of bias | | | | |
| --- | --- | --- | --- | --- | --- |
|  | Confounding | | | | Other domains |
|  | Outcome | Results | Covariates to be controlled | Risk of bias judgement |  |
| Launey 2019 (4) | Incidence of NOAF | RD -11.9% (95% CI: -23.4% to  -0.5%), RR 0.58 (95% CI: 0.35 to 0.98) | Age, sex, preceding cardiovascular disease, acute renal failure, acute respiratory failure, APACHE score, and the use of vasopressors (26) | ***Serious***  Due to missing covariates (sex, preceding cardiovascular disease, and the use of vasopressors) | N/A |
| Walkey 2016 (12) | Mortality | RR 0.99 (95% CI: 0.86 to 1.15), RR 0.75 (95% CI: 0.64 to 0.88), RR 0.67 (95% CI: 0.59 to 0.77) | Sickness score e.g. SOFA or individual components of score | ***Serious***  Although a comprehensive list of relevant covariates was used, it is unclear how many of the variables were measured at baseline (i.e. just prior to treatment). Acute organ failure was recorded at admission.  Reliability or validity of covariate measurement was low enough to expect the possibility of serious residual confounding | N/A |
| Walkey 2016b (13) | Stroke; Bleeding | Stroke: RR 0.85 (95% CI 0.57 to 1.27)  Bleeding: 0.97 (95% CI: 0.83 to 1.14) | For stroke: Age, Sex, Heart failure, Hypertension, Diabetes, Carotid artery disease, Hypercholesterolaemia  For bleeding: ± illness severity, systemic inflammation, type and location of surgery, nutritional status, invasive devices, and acute coagulopathy and thrombocytopaenia | ***Serious for both outcomes***  For stroke: Due to missing covariates (carotid artery disease and hypercholesterolaemia)  For bleeding: Although a comprehensive list of relevant covariates was used, it is unclear how many of the variables were measured at baseline (i.e. just prior to treatment).  Reliability or validity of covariate measurement was low enough to expect the possibility of serious residual confounding | N/A |

# Further summary tables

Supplementary table 12 - Overview of the primary study evidence by intervention and study design

| Intervention | | Study design | | | | |
| --- | --- | --- | --- | --- | --- | --- |
|  |  | RCT | Prospective comparative | Retrospective comparative | Prospective non-comparative studies | Retrospective non-comparative studies |
| Pharmacological treatments | Amiodarone | Delle Karth et al 2001, n=60 | Gerlach et al 2008, n=61 | Walkey et al 2016, n=3174  Cho et al 2017, n=448  Matsumoto et al 2015, n=276  Balik et al 2017, n=234  Mieure et al 2011, n=126  Jaffer et al 2016, n=65  McKenzie Brown et al 2018, n=33 | Sleeswijk et al 2008, n=29  Slavik et al 2003, n= not reported | Liu et al 2016, n=240  Mitric et al 2016, n=177  Kanji et al 2012, n=139  Mayr et al 2004, n=131  Burris et al 2010, n=30 |
|  | Beta blockers | Balser et al 1998, n=55 | No studies | Walkey et al 2016, n=3174  Matsumoto et al 2015, n=276  Balik et al 2017, n=234  Mieure et al 2011, n=126  Jaffer et al 2016, n=65  McKenzie Brown et al 2018, n=33 | Nakamura et al 2016, n=16 | Liu et al 2016, n=240  Kanji et al 2012, n=139  Burris et al 2010, n=30 |
|  | Calcium-channel blockers | Delle Karth et al 2001, n=60  Balser et al 1998, n=55 | Gerlach et al 2008, n=61 | Walkey et al 2016, n=3174  Mieure et al 2011, n=126  Jaffer et al 2016, n=65  McKenzie Brown et al 2018, n=33 | No studies | Liu et al 2016, n=240  Burris et al 2010, n=30 |
|  | Propafenone | No studies | No studies | Balik et al 2017, n=234 | No studies | No studies |
|  | Digoxin | No studies | No studies | Walkey et al 2016, n=3174 | No studies | Liu et al 2016, n=240  Burris et al 2010, n=30 |
|  | Ibutilide | No studies | No studies | No studies | Hennersdorf et al 2002, n=26  Delle Karth et al 2005, n=17 | No studies |
|  | Magnesium sulphate infusion | No studies | No studies | No studies | Sleeswijk et al 2008, n=29 | No studies |
| Prophylactic treatments | Hydrocortisone | No studies | Launey et al 2019, n=261 | Kane and Hanes 2014, n=109 | No studies | No studies |
| Electrical treatments | Direct-current cardioversion (DCC) | No studies | No studies | No studies | Mayr et al 2003, n=37 | No studies |
|  | Electrical cardioversion (ECV) | No studies | No studies | No studies | No studies | Liu et al 2016, n=240  Kyo et al 2019, n=85 |
| Anticoagulants | | No studies | No studies | Walkey et al 2016b, n=7522 | Slavik et al 2003, n= not reported | No studies |

Supplementary table 13 - Characteristics and methods of studies comparing beta blockers and digoxin

| Authors | Sample size and setting | Primary diagnosis | Study design and risk of bias | Intervention | Rate control outcome | Rhythm control outcome | Mortality outcome |
| --- | --- | --- | --- | --- | --- | --- | --- |
| Walkey et al. (2016) | n= 3,174^[[2]](#footnote-3)^ (NOAF patients)  Setting: USA | Sepsis | Retrospective comparative  Risk of bias: Serious | Beta blockers (metoprolol, esmolol, atenolol, labetalol,  propranolol) versus digoxin | Not assessed | Not assessed | Hospital: RR^[[3]](#footnote-4)^ 0.75 (95% CI: 0.64-0.88) |

Supplementary table 14 - Characteristics and methods of studies comparing anticoagulation and no treatment

| Authors | Sample size and setting | Primary diagnosis | Study design and risk of bias | Intervention | Stroke outcome | Bleeding outcome | Mortality outcome |
| --- | --- | --- | --- | --- | --- | --- | --- |
| Walkey et al. (2016)b (13) | n=7522 (NOAF patients)  n=5585 (NOAF patients analysed with propensity score approach)  Setting: USA | Sepsis | Retrospective comparative  Risk of bias: Serious | Anticoagulants (intravenous heparin sodium, subcutaneous enoxaparin sodium,  subcutaneous dalteparin sodium,  fondaparinux sodium, warfarin sodium) versus no treatment | RR^[[4]](#footnote-5)^ 0.85 (95% CI: 0.57 - 1.27) | RR 0.97 (95% CI: 0.83 to 1.14) | Not assessed |

# Summary of reviews, surveys and opinion pieces

## Reviews and guidelines

Twelve review articles (27-38) were included in the current review. Of these, two were systematic reviews (27, 30), eight were narrative review articles (28, 31-33, 36, 38) and four were review articles (29, 34, 35, 37) that proposed a treatment algorithm based on available evidence. Most included reviews (27-29, 31-35, 37, 38) (n=10) were published after 2012. No guidelines were identified in this scoping review.

## Systematic reviews

Yoshida et al. (2015) conducted a systematic review of epidemiology, prevention, and treatment of NOAF in critically ill patients (27). One database was searched, and eligibility criteria were specified for study inclusion in the systematic review. The authors assessed the methodological quality of included studies using the GRADE system. No studies on NOAF prevention were included in the systematic review, and five studies investigating treatments for NOAF were eligible. The five included studies, of which one was an RCT, evaluated the effectiveness of NOAF treatments such as amiodarone, beta blockers, calcium channel blockers, digoxin, magnesium sulphate, and direct current cardioversion. However, no conclusive findings on the treatment strategies were reported. The authors concluded that the current evidence for NOAF management in general ICU population is very limited and further research is urgently needed (27).

In 2008, Kanji et al. published a systematic review of RCTs assessing the treatments of NOAF in noncardiac ICU patients (30). Three databases were systematically searched, study eligibility criteria were clearly specified, and quality assessment of each included RCT was conducted using a basic rating instrument (the Jadad scale). Four RCTs which assessed the efficiency of procainamide, flecainide, esmolol, amiodarone, verapamil, diltiazem, and magnesium were included in the Kanji et al. review. Two RCTs had a study population consisting of <70% NOAF patients (and so were not eligible for this scoping review). The authors were not able to make evidence-based recommendations for pharmacologic rhythm conversion strategies for a general ICU NOAF population due to considerable methodological heterogeneity of the included RCTs. The need for well-designed and adequately powered RCTs to evaluate treatment strategies for critically ill patients with NOAF was emphasised. Moreover, it was recommended that future research should address treatments of choice and goals of care using a standardized outcome measure of success (30).

## Other types of review

The evidence on pharmacological (28, 29, 31-37), and electrical (29, 33, 36, 37) treatment strategies for NOAF was discussed in the other review articles. Four articles (28, 31, 33, 37) discussed the management of NOAF in sepsis patients, and six articles (31, 34-38) reviewed the literature on anticoagulation strategies for critically ill patients with NOAF.

Pharmacological treatments

It was widely reported that management of arrhythmias in critical care settings is a major problem (33, 36) and research on optimal therapeutic strategies for critically-ill patients with NOAF is urgently needed (28, 29, 31-33, 35-37). Some articles (29, 32, 35) argued that beta blockers may be a reasonable first-choice treatment due to the current evidence of decreased mortality (29), and improved heart rate control (29, 32). In contrast, some authors discussed amiodarone as a potentially effective treatment (28, 31, 33, 34, 36) based on current evidence and its widespread use, though it was also recognised that amiodarone has potentially significant side effects (28, 34, 36). Other pharmacological treatments such as propafenone (28, 36), calcium channel blockers (29, 36, 37), digoxin (28, 29, 36), and ibutilide (36) were discussed though no conclusive findings were made. Four articles (29, 34, 35, 37) proposed a treatment algorithm but the algorithms should be interpreted cautiously as they were developed based on limited evidence which was not identified and critiqued systematically.

Electrical treatments

Reviews suggested direct current cardioversion might often be unsuccessful (29) and might be also associated with high relapse rate (29, 36). More evidence in critically ill populations is needed to support this (36), and the current findings should be used to guide research in therapy and mechanisms.

Anticoagulants

A review article (36) concluded that there was no clear evidence whether stroke risk reduction outweighs the increased risk of bleeding when using therapeutic anticoagulation in critically ill patients with NOAF. Labbe et al. (2015) reported a high frequency of major bleeding events and recommended to administer anticoagulation therapy only in patients with the highest risk of arterial thromboembolic events (38). This assertion of high bleeding rates referenced one study that did not compare bleeding events between patients that received anticoagulation and those that did not, and one study that found no significant difference in bleeding events between these two groups. A patient-centred single-case decision approach whether to use anticoagulant therapy was also suggested in another review (35). Sibley and Muscedere (2015) recommended initiating anticoagulation if AF persists for more than 48 hours, and in patients with high risk of arterial thromboembolic events (35). Only one review article (35) discussed which drug would be appropriate to use for anticoagulation in ICU patients. Unfractionated heparin was reported to be a drug of choice for critically-ill patients due to its short half-life and reversibility with protamine; however, it must be noted that the authors of the review did not provide any references for this statement (35).

## Surveys and opinion pieces

### Surveys

Only one survey (39) was identified in the current review. The UK-wide survey on the practice of the management of NOAF in critically ill patients was conducted in 2016 and was sent to all members of the Intensive Care Society. Three thousand one hundred fifty-two questionnaires were sent, and 397 responses were received. The survey included questions on demographic variables of participants and their critical care unit, and a set of questions aimed to determine the management strategies for NOAF and anticoagulation practice. Seventy-two percent of respondents were consultants, 46% worked in a District General Hospital, and 81% worked in a mixed ICU. The authors reported that 81% of respondents used amiodarone for the treatment of NOAF. Only 12% of respondents reported using beta-blockers. It was reported that 64% of respondents would not use anticoagulant therapy in critically ill patients with NOAF, while 31% of respondents would start anticoagulation within 72 hours. The survey revealed that low or high molecular weight heparin was considered appropriate for anticoagulant therapy (39). The limitations of this survey should be considered. Firstly, it was sent only to the members of the Intensive Care Society and this cohort represents two thirds of practicing intensivists in the UK. And secondly, the survey had a very low response rate of 13%, indicating that the results might not represent current practice in the UK.

### Opinion pieces

Four opinion pieces (40-43) were identified in the current review. Two (42, 43) were published in 2008 and are responses to a systematic review of RCTs investigating treatments for NOAF in critically-ill non cardiac ICU population (30). Both authors agree with the conclusion by Kanji et al. (2008) – the evidence is lacking, and the answers still need to be provided. Walton (2008) believes that the best agent for use in NOAF is amiodarone as it combines rapid rate control effects and a low risk of precipitating ventricular tachyarrhythmias (42). However, Trohman (2008) favours the use of beta-blockers as the initial pharmacotherapy (43). Both authors (42, 43) emphasised that treatments must be carefully studied to design an evidence-based approach to guide treatment strategies in NOAF patients on an ICU.

Walkey et al. (2015) recommends beta-blockers as a reasonable first choice of initial AF therapy, given the limited and indirect evidence (40). The authors also commented on managing risks of stroke and concluded that evidence is currently lacking on risks of bleeding and estimates of stroke risk reduction associated with use of anticoagulation in critically ill patients. Therefore, the authors did not recommend using anticoagulation in NOAF patients with elevated bleeding risk as it is not currently known whether the benefits outweigh the risks (40).

Vieillard‑Baron and Boyd (2017) suggest a non-antiarrhythmic-based approach to reduce NOAF by optimising electrolytes and fluid status, limitation of sympathetic activation, and control of the central venous catheter position before considering any anti-arrhythmic drugs (41). It must be noted that this treatment strategy was developed by the authors and was based on pathophysiology of AF and its risk factors present in patients with sepsis. No high-quality evidence is available to support this treatment approach.

# Excluded studies

## Excluded studies on full text with reason for exclusion

| Study | Excluded based on | | | |
| --- | --- | --- | --- | --- |
|  | Population | Study design | Outcome | Intervention |
| *AACN Bold Voices* (2018) |  | X |  |  |
| Agnihotri et al. (2017) | X |  |  |  |
| Akella et al. (2017) | X |  |  |  |
| Akhtar et al. (2011) | X |  |  |  |
| Al-Hashimi et al. (2012) | X |  |  |  |
| Al-Khafaji et al. (2006) |  | X |  |  |
| Ambrus et al. (2015) |  |  |  | X |
| Anane et al. (2011) |  |  | X |  |
| Ando et al. (2004) | X |  |  |  |
| Arita et al. (2017) |  | X |  |  |
| Arnautovic et al. (2018) | X |  |  |  |
| Arrigo et al. (2014) |  | X |  |  |
| Arrigo et al. (2015) | X |  |  |  |
| Arrigo et al. (2016) |  | X |  |  |
| Arrigo et al. (2018) |  |  |  | X |
| Arrigo et al. (2018) |  |  |  | X |
| Arsura et al. (1987) | X |  |  |  |
| Aydogdu et al. (2017) | X |  |  |  |
| Badheka et al. (2012) | X |  |  |  |
| Balik (2018) | X |  |  |  |
| Balik et al. (2016) |  |  | X |  |
| Barranco et al. (1994) | X |  |  |  |
| Bender et al. (1996) |  |  | X |  |
| Bernal et al. (2018) |  |  |  | X |
| Bernard et al. (2003) | X |  |  |  |
| Bowles et al. (1990) | X |  |  |  |
| Carrera et al. (2016) |  |  |  | X |
| Champion (2015) | X |  |  |  |
| Champion (2017) |  | X |  |  |
| Champion et al. (2018) | X |  |  |  |
| Chapman et al. (1993) | X |  |  |  |
| Clayton et al. (2018) |  |  |  | X |
| Clemo et al. (1998) | X |  |  |  |
| Crawford et al. (2007) | X |  |  |  |
| Darwish et al. (2011) | X |  |  |  |
| Darwish et al. (2013) | X |  |  |  |
| Davies et al. (1992) | X |  |  |  |
| Delle Karth et al. (2003) |  |  |  | X |
| Duarte et al. (2017) |  |  |  | X |
| Duarte et al. (2019) |  | X |  |  |
| Duby et al. (2017) |  |  |  | X |
| Eckardt (2014) |  | X |  |  |
| Edwards et al. (1986) | X |  |  |  |
| Edwards et al. (1987) | X |  |  |  |
| Faniel et all. (1981) | X |  |  |  |
| Flato et al. (2011) | X |  |  |  |
| Friedman et al. (1991) | X |  |  |  |
| Fu et al. (2018) |  |  |  | X |
| Gandhi et al. (2015) |  |  | X |  |
| Ganguly et al. (2015) |  |  |  | X |
| Gibb et al. (2018) |  | X |  |  |
| Goodman et al. (2008) | X |  |  |  |
| Gray (1988) | X |  |  |  |
| Gray et al. (2016) | X |  |  |  |
| Guenancia et al. (2016) |  |  |  | X |
| Guenancia et al. (2018) |  |  |  | X |
| Guha et al. (2019) |  |  |  | X |
| Guillot et al. (2009) |  | X |  |  |
| Hadjizacharia et al. (2011) | X |  |  |  |
| Hampden-Martin et al. (2018) |  |  | X |  |
| Harazim et al. (2017) |  | X |  |  |
| Hayashi et al. (2005) | X |  |  |  |
| Heinz (2006) | X |  |  |  |
| Heinz (2008) |  |  |  | X |
| Heinz (2013) |  | X |  |  |
| Hilkens et al. (2009) |  |  | X |  |
| Holt (1989) | X |  |  |  |
| Hughes et al. (2000) | X |  |  |  |
| Iberti et al. (1986) | X |  |  |  |
| Ito et al. (2006) | X |  |  |  |
| Jelliffe (2016) |  |  |  | X |
| Jenkins et al. (2016) |  | X |  |  |
| Kanjanahattakij et al. (2018) |  |  |  | X |
| Karth et al. (2000) |  | X |  |  |
| Kerton et al. (2018) | X |  |  |  |
| Khoguli et al. (1994) | X |  |  |  |
| Khoury et al. (2018) |  | X |  |  |
| Klein Klouwenberg et al. (2017) |  |  | X |  |
| Krumpl et al. (2012) | X |  |  |  |
| Kumar (1996) | X |  |  |  |
| Labakis et al. (2013) |  |  |  | X |
| Leelathanalerk et al. (2017) |  |  | X |  |
| Lewis et al. (2016) |  |  |  | X |
| Lim et al. (2004) | X |  |  |  |
| Makrygiannis et al. (2014) |  |  | X |  |
| Malik et al. (2013) | X |  |  |  |
| Marik et al. (2000) | X |  |  |  |
| Marinheiro et al. (2016) |  |  | X |  |
| Marque et al. (2012) |  | X |  |  |
| Meierhenrich et al. (2010) |  |  | X |  |
| Miller et al. (2018) |  | X |  |  |
| Mirhoseini et al. (2018) | X |  |  |  |
| Moran et al. (1995) | X |  |  |  |
| Moskowitz et al. (2016) | X |  |  |  |
| Moss et al. (2016) |  |  |  | X |
| Murcia et al. (1995) | X |  |  |  |
| Nadler (2016) | X |  |  |  |
| Nair et al. (2007) | X |  |  |  |
| Nakos et al. (2015) | X |  |  |  |
| Nallapareddy et al. (2010) |  |  | X |  |
| Needleman et al. (2017) |  |  |  | X |
| Niazi et al. (2017) |  |  |  | X |
| Nietupski et al. (2014) |  |  | X |  |
| Nitti et al. (2018) | X |  |  |  |
| Nowakowski (1992) | X |  |  |  |
| Okajima et al. (2015) | X |  |  |  |
| Papathanasiou et al. (2015) | X |  |  |  |
| Personett et al. (2014) | X |  |  |  |
| Philip et al. (2010) |  |  |  | X |
| Pinski (2004) |  |  | X |  |
| Poveda-Jaramillo et al. (2018) | X |  |  |  |
| Pravinkumar (2003) |  | X |  |  |
| Pye et al. (1994) | X |  |  |  |
| Quasim et al. (2010) |  |  |  | X |
| Quon et al. (2018) | X |  |  |  |
| Ratnaparkhi et al. (2010) |  |  | X |  |
| Richards et al. (2006) | X |  |  |  |
| Rudiger et al. (2013) | X |  |  |  |
| Salem et al. (2016) | X |  |  |  |
| Salem et al. (2017) | X |  |  |  |
| Salerno (1992) | X |  |  |  |
| Salman et al. (2006) |  |  | X |  |
| Samarin et al. (2015) | X |  |  |  |
| Sanai et al. (1993) | X |  |  |  |
| Sargent (1999) | X |  |  |  |
| Schoaps et al. (2016) |  |  | X |  |
| Seemann et al. (2015) |  |  | X |  |
| Seguin et al. (2006) |  |  | X |  |
| Sekhri et al. (2009) | X |  |  |  |
| Sekhri et al. (2009) | X |  |  |  |
| Sen et al. (2015) | X |  |  |  |
| Shah et al. (2014) |  |  |  | X |
| Shaver et al. (2015) |  |  |  | X |
| Shibata et al. (2016) | X |  |  |  |
| Smith et al. (2018) | X |  |  |  |
| Thomas T et al. (2018) |  |  | X |  |
| Tober et al. (2012) |  |  |  | X |
| Tracy et al. (2014) | X |  |  |  |
| Trappe (2010) | X |  |  |  |
| Trappe et al. (2003) | X |  |  |  |
| Trohman (2000) | X |  |  |  |
| Trottier et al. (2012) |  |  | X |  |
| Van der Does et al. (2018) | X |  |  |  |
| Varriale at al. (2000) | X |  |  |  |
| Walkey et al. (2014) |  |  |  | X |
| Walkey et al. (2015) | X |  |  |  |
| Walkey et al. (2017) |  |  |  | X |
| Willich et al. (2012) |  | X |  |  |
| Wong et al. (2012) | X |  |  |  |
| Wood et al. (2009) | X |  |  |  |
| Yokota et al. (2018) |  |  |  | X |
| Yoshida et al. (2018) |  |  | X |  |
| Yucel et al. (2015) |  |  |  | X |
| Zochios et al. (2013) |  | X |  |  |

## List of excluded studies on full text

AACN: Preventing New-Onset Atrial Fibrillation in the ICU. AACN Bold Voices 2018; 10(8):20-20

Agnihotri K, Patel P, Charilou P, Patel NJ, et al: Impact of atrial fibrillation on mortality, length of stay and cost in patients with sepsis. 38th Annual Scientific Sessions of the Heart Rhythm Society, Heart Rhythm 2017 United States 2017; 14(5 Supplement 1):S409-S410

Akella K, Akella S, Akella SL, Chendrasekhar A: Atrial fibrillation in elderly (age > 65 years) trauma patients is associated with increased mortality and morbidity. CHEST 2017 Annual Meeting Canada 2017; 152(4 Supplement 1):A66

Akhtar MI, Ullah H, Hamid M: Magnesium, a drug of diverse use. Journal of the Pakistan Medical Association 2011; 61(12):1220-1225

Al-Hashimi M, Thompson JP: Drugs acting on the heart: Anti-arrhythmics. Anaesthesia and Intensive Care Medicine 2012; 13(8):374-377

Al-Khafaji A, Cho Su M: Atrial fibrillation in critical care. [Comment on: Intensive Care Med 2006 Mar;32(3):398-404; PMID: 16496203 [https://wwwncbinlmnihgov/pubmed/16496203]] 2006; 32(7):1099-1100

Ambrus Daniel B, Benjamin Emelia J, Bajwa Ednan K, Hibbert Kathryn A, et al: Risk factors and outcomes associated with new-onset atrial fibrillation during acute respiratory distress syndrome. Journal of Critical Care 2015; 30(5):994-997

Anane C, Owusu IK, Attakorah J: Monitoring amiodarone therapy in cardiac arrthythmias in the intensive care unit of a teaching hospital in Ghana. Internet Journal of Cardiology 2011; 10(1)

Ando G, Di Rosa S, Rizzo F, Carerj S, et al: Ibutilide for cardioversion of atrial flutter: Efficacy of a single close in recent-onset arrhythmias. Minerva Cardioangiologica 2004; 52(1):37-42

Arita Y, Segawa T, Yamamoto S, Hasegawa S: Landiolol is effective for the treatment of tachycardia-induced cardiogenic shock in patients during septic shock therapy. BMJ case reports 2017; 2017

Arnautovic J, Mazhar A, Souther B, Mikhjian G, et al: New-onset atrial fibrillation in patients with septic shock. 47th Society of Critical Care Medicine Critical Care Congress, SCCM 2018 United States 2018; 46(Supplement 1):74

Arrigo M, Bettex D, Rudiger A: Management of atrial fibrillation in critically ill patients. [Comment in: Crit Care Res Pract 2016;2016:8985161; PMID: 27641992 [https://wwwncbinlmnihgov/pubmed/27641992]][Comment in: Crit Care Res Pract 2016;2016:9724504; PMID: 27642081 [https://wwwncbinlmnihgov/pubmed/27642081]] 2014; 2014:840615

Arrigo M, Bettex D, Rudiger A: Treatment of atrial fibrillation in intensive care units and emergency departments. Behandlung von Vorhofflimmern in der Intensiv- und Notfallmedizin 2015; 110(8):614-620

Arrigo M, Bettex D, Rudiger A: Response to: Comment on "Management of Atrial Fibrillation in Critically Ill Patients". [Comment on: Crit Care Res Pract 2014;2014:840615; PMID: 24527212 [https://wwwncbinlmnihgov/pubmed/24527212]] 2016; 2016:9724504

Arrigo M, Feliot E, Gayat E, Mebazaa A: Cardiovascular events after ICU discharge in patients with new-onset atrial fibrillation: A report from the FROG-ICU study. [Comment on: Int J Cardiol 2018 Nov 1;270:189; PMID: 30219534 [https://wwwncbinlmnihgov/pubmed/30219534]][Comment on: Int J Cardiol 2018 Sep 1;266:95-99; PMID: 29887481 [https://wwwncbinlmnihgov/pubmed/29887481]] 2018; 270:203

Arrigo M, Ishihara S, Feliot E, Rudiger A, et al: New-onset atrial fibrillation in critically ill patients and its association with mortality: A report from the FROG-ICU study. [Comment in: Int J Cardiol 2018 Sep 1;266:147-148; PMID: 29887432 [https://wwwncbinlmnihgov/pubmed/29887432]][Comment in: Int J Cardiol 2018 Nov 1;270:189; PMID: 30219534 [https://wwwncbinlmnihgov/pubmed/30219534]][Comment in: Int J Cardiol 2018 Nov 1;270:203; PMID: 30045823 [https://wwwncbinlmnihgov/pubmed/30045823]] 2018; 266:95-99

Arsura EL, Solar M, Lefkin AS, Scher DL, et al: Metoprolol in the treatment of multifocal atrial tachycardia. Critical care medicine 1987; 15(6):591-594

Aydogdu M, Hanazay C, Aldag Y, Baha A, et al: Effects of Atrial Fibrillation on Intensive Care Unit Outcomes in Patients with Respiratory Failure. JOURNAL OF MEDICAL AND SURGICAL INTENSIVE CARE MEDICINE 2017; 8:32-38

Badheka AO, Tuliani T, Rathod A, Shenoy M, et al: Role of lipid lowering therapy and renin angiotensin blockade in outcomes of patients with atrial fibrillation. American Journal of Cardiology 2012; 109(8):1238

Balik M: New-onset atrial fibrillation in critically ill patients - Implications for rhythm rather than rate control therapy? INTERNATIONAL JOURNAL OF CARDIOLOGY 2018; 266:147-148

Balik M, Kolnikova I, Maly M, Waldauf P, et al: Antiarrhythmic therapy for supraventricular arrhythmias in septic shock. 29th Annual Congress of the European Society of Intensive Care Medicine, ESICM 2016 Italy 2016; 4(Supplement 1)

Barranco F, Sanchez M, Rodriguez J, Guerrero M: Efficacy of flecainide in patients with supraventricular arrhythmias and respiratory insufficiency. Intensive care medicine 1994; 20(1):42-44

Bender JS: Supraventricular tachyarrhythmias in the surgical intensive care unit: an under-recognized event. The American surgeon 1996; 62(1):73-75

Bernal E, Wolf S, Cripps M: New-onset, postoperative tachyarrhythmias in critically ill surgical patients. BURNS 2018; 44:249-255

Bernard EO, Schmid ER, Schmidlin D, Scharf C, et al: Ibutilide versus amiodarone in atrial fibrillation: A double-blinded, randomized study. CRITICAL CARE MEDICINE 2003; 31:1031-1034

Bowles HF, Thangathurai D, Morgan GE, Mikhail M: Management of supraventricular tachycardia in septic patients. Anaesthesia 1990; 45(9):787-788

Carrera P, Thongprayoon C, Cheungpasitporn W, Iyer Vivek N, et al: Epidemiology and outcome of new-onset atrial fibrillation in the medical intensive care unit. Journal of critical care 2016; 36:102-106

Champion S: Comment on "management of atrial fibrillation in critically ill patients". Critical care research and practice 2015; 2015:732598

Champion S: An Overlook of New-Onset Atrial Fibrillation in the Critically Ill Using Automated Detection: Have We Over Looked at It? Critical Care Medicine 2017; 45(11)

Champion S, Gauzere BA, Vandroux D, Lefort Y: [Is it worth delivering Direct-Current Counter shock to critically ill patients with supra-ventricular tachyarrhythmia?]. Annales de cardiologie et d'angeiologie 2018; 67(4):260-263

Chapman MJ, Moran JL, O'Fathartaigh MS, Peisach AR, et al: Management of atrial tachyarrhythmias in the critically ill: a comparison of intravenous procainamide and amiodarone. Intensive care medicine 1993; 19(1):48-52

Clayton B, Ball S, Read J, Waddy S: Risk of thromboembolism in patients developing critical illness-associated atrial fibrillation. Clinical medicine (London, England) 2018; 18(4):282-287

Clemo HF, Wood MA, Gilligan DM, Ellenbogen KA: Intravenous Amiodarone for acute heart rate control in the critically ill patient with atrial tachyarrhythmias. AMERICAN JOURNAL OF CARDIOLOGY 1998; 81:594-598

Crawford TC, Oral H: Cardiac Arrhythmias: Management of Atrial Fibrillation in the Critically Ill Patient. Critical Care Clinics 2007; 23(4):855-872

Darwish OS, Strube S, Phan A, Tanios M: The safety and efficacy of anticoagulation for atrial fibrillation in patients with severe sepsis in the medical intensive care unit. Quality of Care and Outcomes Research in Cardiovascular Disease and Stroke 2010 Scientific Sessions, QCOR 2010 Washington, DC United States 2011; 4(6 MeetingAbstracts2010)

Darwish Omar S, Strube S, Nguyen Huan M, Tanios Maged A: Challenges of Anticoagulation for Atrial Fibrillation in Patients With Severe Sepsis. ANNALS OF PHARMACOTHERAPY 2013; 47:1266-1271

Davies GE, Cudworth P, Lawler PG: Intravenous magnesium therapy in critically ill patients [18]. Anaesthesia 1992; 47(12):1104

Delle K, Georg, Reinelt P, Buberl A, et al: Circadian variation in ventricular tachycardia and atrial fibrillation in a medical-cardiological ICU. Intensive care medicine 2003; 29(6):963-968

Duarte Péricles AD, Leichtweis Gustavo E, Andriolo L, Delevatti Yasmim A, et al: Factors Associated with the Incidence and Severity of New-Onset Atrial Fibrillation in Adult Critically Ill Patients. Critical Care Research and Practice 2017; 2017:1-8

Duarte Péricles AD, Leichtweis Gustavo E, Andriolo L, Delevatti Yasmim A, et al: Corrigendum to "Factors Associated with the Incidence and Severity of New-Onset Atrial Fibrillation in Adult Critically Ill Patients". Critical Care Research & Practice 2019:1-1

Duby Jeremiah J, Heintz Shannon J, Bajorek Sarah A, Heintz Brett H, et al: Prevalence and Course of Atrial Fibrillation in Critically Ill Trauma Patients. Journal of intensive care medicine 2017; 32(2):140-145

Eckardt L: Innovations to the Therapy of Atrial Fibrillations in Intensive Care Medicine. MEDIZINISCHE KLINIK-INTENSIVMEDIZIN UND NOTFALLMEDIZIN 2014; 109:564-565

Edwards JD, Kishen R: Significance and management of intractable supraventricular arrhythmias in critically ill patients. Critical Care Medicine 1986; 14(4):280-282

Edwards JD, Wilkins RG: Atrial fibrillation precipitated by acute hypovolaemia. British medical journal (Clinical research ed) 1987; 294(6567):283-284

Faniel R, Schoenfeld P: Intravenous amiodarone: A successful treatment for rapid atrial fibrillation in intensive care patients. European Heart Journal 1981; 2(Suppl.A):115

Flato Uri Adrian P, Buhatem T, Merluzzi T, Bianco Antonio Carlos M: New anticoagulants in critical care settings. Novos anticoagulantes em cuidados intensivos 2011; 23(1):68-77

Friedman HZ, Goldberg SF, Bonema JD, Cragg DR, et al: Acute complications associated with new-onset atrial fibrillation. [Comment in: Am J Cardiol 1992 Mar 15;69(8):842; PMID: 1546679 [https://wwwncbinlmnihgov/pubmed/1546679]] 1991; 67(5):437-439

Fu J, Bi H, Xia Y, Fang H, et al: [Risk factors of atrial fibrillation in critical ill patients]. Zhonghua wei zhong bing ji jiu yi xue 2018; 30(4):337-341

Gandhi S, Litt D, Narula N: New-onset atrial fibrillation in sepsis is associated with increased morbidity and mortality. NETHERLANDS HEART JOURNAL 2015; 23:82-88

Ganguly S, Brown T, Pritchett C, Edie S, et al: Atrial fibrillation in intensive care. Intensive Care Medicine Experimental 2015; 3(Supplement 1):A209

Gibb S, Rehberg S: The role of the ultra short-acting beta1-adrenoreceptor antagonist Landiolol in the treatment of atrial fibrillation: Pharmacology, clinical application and current evidence in anaesthesiology, intensive care and emergency medicine. Rolle des ultrakurzwirksamen beta1-Adrenorezeptor-Antagonisten Landiolol in der Therapie des Vorhofflimmerns: Pharmakologie, klinische Anwendung und derzeitige Evidenz in der Anasthesiologie, Intensiv- und Notfallmedizin 2018; 59(7-8):407-421

Goodman S, Weiss Y, Weissman C: Update on cardiac arrhythmias in the ICU. Current Opinion in Critical Care 2008; 14(5):549-554

Gray RJ: Managing critically ill patients with esmolol. An ultra short-acting beta-adrenergic blocker. Chest 1988; 93(2):398-403

Gray J, Haydock P, Wong A, Pierce JMT: Cardiac arrhythmias in the critically ill. ANAESTHESIA AND INTENSIVE CARE MEDICINE 2016; 17:38-47

Guenancia C, Laurent G, Bruyère R, Quenot J-P: New-Onset Atrial Fibrillation in Sepsis: So Common, but So Different. Critical Care Medicine 2016; 44(5)

Guenancia C, Dargent A, Large A, Andreu P, et al: New-onset atrial fibrillation in ICU: A FROG in the throat. [Comment on: Int J Cardiol 2018 Sep 1;266:95-99; PMID: 29887481 [https://wwwncbinlmnihgov/pubmed/29887481]][Comment in: Int J Cardiol 2018 Nov 1;270:203; PMID: 30045823 [https://wwwncbinlmnihgov/pubmed/30045823]] 2018; 270:189

Guha K, Clayton B: Atrial fibrillation...Clayton B , Ball S , Read J , Waddy S . Risk of thromboembolism in patients developing critical illness associated atrial fibrillation . Clin Med 2018 ; 18 : 282 – 7. Clinical Medicine 2019; 19(1):90-90

Guillot M, Diouf M, Harlay ML, Janssen-Langenstein R, et al: Supraventricular rhythm disorders of the rate/rhythm in critically ill patients. Les troubles du rythme supraventriculaires en reanimation 2009; 18(3):246-253

Hadjizacharia P, O'Keeffe T, Brown Carlos VR, Inaba K, et al: Incidence, risk factors, and outcomes for atrial arrhythmias in trauma patients. The American surgeon 2011; 77(5):634-639

Hampden-Martin A, Miller N, Johnston B, Welters I: A retrospective observational cohort study of patients admitted to a mixed medical and surgical intensive care unit in the United Kingdom with atrial fibrillation: Incidence, risk factors and treatment efficacy. 31st European Society of Intensive Care Medicine Annual Congress, ESICM 2018 France 2018; 6(Supplement 2)

Harazim M, Karvunidis T, Radej J, Horak J, et al: Atrial fibrillation in critically ill patients. Fibrilace sini u kriticky nemocnych 2017; 28(4):248-254

Hayashi M, Tanaka K, Kato T, Morita N, et al: Enhancing electrical cardioversion and preventing immediate reinitiation of hemodynamically deleterious atrial fibrillation with class III drug pretreatment. Journal of cardiovascular electrophysiology 2005; 16(7):740-747

Heinz G: Atrial fibrillation in the intensive care unit. INTENSIVE CARE MEDICINE 2006; 32:345-348

Heinz G: Arrhythmias in the ICU: What do we know? AMERICAN JOURNAL OF RESPIRATORY AND CRITICAL CARE MEDICINE 2008; 178:1-2

Heinz G: [Atrial fibrillation in the ICU. Distinct entity--special treatment?]. Vorhofflimmern auf der Intensivstation Eigene Entitat--spezielle Behandlung? 2013; 108(7):549-554

Hilkens M, Pickkers P, Peters WH, van der Hoeven JG, et al: No elevation of glutathione S-transferase-a1-1 by amiodarone loading in intensive care unit patients with atrial fibrillation. Anaesthesia & Intensive Care 2009; 37(2):281-285

Holt AW: Hemodynamic responses to amiodarone in critically ill patients receiving catecholamine infusions. Critical care medicine 1989; 17(12):1270-1276

Hughes M, Binning A: Intravenous amiodarone in intensive care. Time for a reappraisal? Intensive care medicine 2000; 26(12):1730-1739

Iberti TJ, Benjamin E, Paluch TA, Gentili DR, et al: Use of constant-infusion verapamil for the treatment of postoperative supraventricular tachycardia. Critical care medicine 1986; 14(4):283-284

Ito H, Sobue K, So M, Hirate H, et al: Use of landiolol in the perioperative management of supraventricular tachycardia [2]. Journal of Anesthesia 2006; 20(3):253-254

Jelliffe R: Comment on "Management of Atrial Fibrillation in Critically Ill Patients". Critical Care Research & Practice 2016:1-2

Jenkins SA, Griffin R: New-onset atrial fibrillation in intensive care: Incidence, management and outcome. 36th International Symposium on Intensive Care and Emergency Medicine Brussels Belgium 2016; 20(SUPPL. 2)

Kanjanahattakij N, Rattanawong P, Krishnamoorthy P, Horn B, et al: New-onset atrial fibrillation is associated with increased mortality in critically ill patients: a systematic review and meta-analysis. Acta cardiologica 2018:1-8

Karth GD, Geppert A, Haumer M, Neunteufl T, et al: Tachycardial atrial fibrillation and atrial flutter in intensive-care patients: Comparison of diltiazem and amiodarone for control of heart rate - Preliminary results. Tachykardes vorhofflimmern und vorhofflatern bei intensivpatienten: Vergleich von diltiazem und amiodaron zur herzfrequenzkontrolle - Vorlaufige ergebnisse 2000; 7(1):50

Kerton M, Wiggins J, Purkiss M: Cardiac arrhythmias in the critically ill. ANAESTHESIA AND INTENSIVE CARE MEDICINE 2018; 19:298-307

Khoguli SS, Hopkinson RB, Beattie JM, Parmar M, et al: Supraventricular tachydysrhythmias in the ICU [1]. British Journal of Intensive Care 1994; 4(2):44

Khoury J, Azzam ZS: Propafenone for supraventricular arrhythmias in septic shock-Comparison to amiodarone and metoprolol. Journal of Critical Care 2018; 45:247

Klein K, Peter MC, Frencken Jos F, Kuipers S, et al: Incidence, Predictors, and Outcomes of New-Onset Atrial Fibrillation in Critically Ill Patients with Sepsis. A Cohort Study. [Comment in: Am J Respir Crit Care Med 2017 Jan 15;195(2):152-154; PMID: 28084820 [https://wwwncbinlmnihgov/pubmed/28084820]] 2017; 195(2):205-211

Krumpl G, Domanovits H, Stix G, Heinz G, et al: Esmolol in cardiology, emergency and critical-care medicine. Esmolol in kardiologie, notfall- und intensivmedizin 2012; 19(SUPPL. A):2-8

Kumar A: Intravenous amiodarone for therapy of atrial fibrillation and flutter in critically ill patients with severely depressed left ventricular function. Southern medical journal 1996; 89(8):779-785

Labakis M, Makrygiannis S, Margariti A, Rizikou D, et al: Prognostic impact of new-onset atrial fibrillation in intensive care unit patients. 26th Annual Congress of the European Society of Intensive Care Medicine, ESICM 2013 Paris France 2013; 39(SUPPL. 2):S401

Leelathanalerk A, Dongtai W, Huckleberry Y, Kopp B, et al: Evaluation of Deprescribing Amiodarone After New-Onset Atrial Fibrillation in Critical Illness. AMERICAN JOURNAL OF MEDICINE 2017; 130:864-866

Lewis O D, Ngwa J, Gillum Richard F, Thomas A, et al: Incidence, Risk Factors and Outcomes of New Onset Supraventricular Arrhythmias in African American Patients with Severe Sepsis. Ethnicity & Disease 2016; 26(2):205-212

Lim Hoong S, Hamaad A, Lip Gregory YH: Clinical review: clinical management of atrial fibrillation - rate control versus rhythm control. Critical care (London, England) 2004; 8(4):271-279

Makrygiannis SS, Margariti A, Rizikou D, Lampakis M, et al: Incidence and predictors of new-onset atrial fibrillation in noncardiac intensive care unit patients. Journal of Critical Care 2014; 29(4):697

Malik A, Candilio L, Hausenloy DJ: Atrial fibrillation in the intensive care setting. Journal of the Intensive Care Society 2013; 14(2):141-149

Marik PE, Zaloga GP: The management of atrial fibrillation in the ICU. Journal of Intensive Care Medicine 2000; 15(4):181-190

Marinheiro AR, Amador P, Ribeiro R, Alves I, et al: Incidence, management and impact of atrial fibrillation in septic shock. Acute Cardiovascular Care 2016 Portugal 2016; 5(Supplement 1):60-61

Marque S, Launey Y: Treatment of atrial fibrillation in ICU (apart from anticoagulation). Traitement de la fibrillation atriale en reanimation (hors anticoagulation) 2012; 21(2):180-187

Meierhenrich R, Steinhilber E, Eggermann C, Weiss M, et al: Incidence and prognostic impact of new-onset atrial fibrillation in patients with septic shock: A prospective observational study. Critical Care 2010; 14:R108-R108

Miller N, Hampden-Martin A, Johnston B, Welters I: Retrospective observational cohort study of anticoagulation practices, thromboembolic events and bleeding events in patients that develop atrial fibrillation admitted to a mixed surgical and medical ICU in the United Kingdom. 31st European Society of Intensive Care Medicine Annual Congress, ESICM 2018 France 2018; 6(Supplement 2)

Mirhoseini MF, Hamblin SE, Moore WP, Pouliot J, et al: Antioxidant supplementation and atrial arrhythmias in critically ill trauma patients. The Journal of surgical research 2018; 222:10-16

Moran JL, Gallagher J, Peake SL, Cunningham DN, et al: PARENTERAL MAGNESIUM-SULFATE VERSUS AMIODARONE IN THE THERAPY OF ATRIAL TACHYARRHYTHMIAS - A PROSPECTIVE, RANDOMIZED STUDY. CRITICAL CARE MEDICINE 1995; 23:1816-1824

Moskowitz A, Chen K, Cooper A, Chahin A, et al: Management Of Atrial Fibrillation With Rapid Ventricular Response In The Intensive Care Unit: A Secondary Analysis Of Electronic Health Record Data. AMERICAN JOURNAL OF RESPIRATORY AND CRITICAL CARE MEDICINE 2016; 193

Moss TJ, Ruminski C, Lake DE, Calland JF, et al: The impact of incident atrial fibrillation in the intensive care unit. 65th Annual Scientific Session of the American College of Cardiology and i2 Summit: Innovation in Intervention, ACC16 Chicago, IL United States 2016; 67(13 SUPPL. 1):2358

Murcia Llacer B, Broch Porcar MJ, Olivares Toledo D, Alvarez Cebrian F, et al: Intravenous amiodarone in the treatment of supraventricular tachyarrhytmias in critically ill patients with different diseases. AMIODARONA POR VIA INTRAVENOSA EN EL TRATAMIENTO DE LAS TAQUIARRITMIAS SUPRAVENTRICULARES EN PACIENTES CRITICOS CON DISTINTAS PATOLOGIAS 1995; 19(3):118-124

Nadler S: What is the Best Rate Control Agent for Patients with Sepsis and Atrial Fibrillation? Critical Care Alert 2016; 24(1):4-5

Nair GM, Morillo CA: Magnesium in the acute management of atrial fibrillation: Noise or music? Polskie Archiwum Medycyny Wewnetrznej 2007; 117(10):446-447

Nakos G, Roustanis E, Zikou X, Saranti M, et al: Prevalence of atrial fibrillation in the Intensive Care Unit (ICU) setting and challenges in its management. An observational, prospective, single-center study. American Thoracic Society International Conference, ATS 2015 Denver, CO United States 2015; 191(MeetingAbstracts)

Nallapareddy S, Naik M, Nortje J: AUDIT ON MANAGEMENT OF NEW ONSET ATRIAL FIBRILLATION IN GENERAL INTENSIVE CARE UNIT AT UNIVERSITY TEACHING HOSPITAL. INTENSIVE CARE MEDICINE 2010; 36:S123-S123

Needleman JS, Yeh DD, Belcher DM, Quraishi SA: Vitamin D status is associated with new-onset atrial fibrillation in critically ill surgical patients. Clinical Nutrition Week , CNW 2017 United States 2017; 41(2):279-280

Niazi OT, Alkhalil A, Olusanya A, Jain S, et al: Comparison of in-hospital outcomes of septic shock patients with and without atrial fibrillation: A retrospective propensity-matched analysis. 66th Annual Scientific Session of the American College of Cardiology and i2 Summit: Innovation in Intervention, ACC17 United States 2017; 69(11 Supplement 1):548

Nietupski R, Bellamy C, Miano T, Mikkelsen M, et al: Continuation of amiodarone at discharge for new-onset atrial fibrillation in critically ill patients. Critical Care Congress 2015 Phoenix, AZ United States 2014; 42(12 SUPPL. 1):A1403

Nitti C, Falsetti L, Rucco M, Gentili T, et al: CHA2DS2-VASc and HAS-BLED scores do not predict NVAF-related events in a population of critically ill patients. 23 Congresso Nazionale della Societa Scientifica FADOI Italy 2018; 12(2 Supplement 1):89

Nowakowski PA: Supraventricular tachyarrhythmias in the intensive care unit. [Comment on: Postgrad Med 1992 May 1;91(6):293-6, 299-303, 307-10; PMID: 1349745 [https://wwwncbinlmnihgov/pubmed/1349745]] 1992; 92(4):34

Okajima M, Takamura M, Taniguchi T: Landiolol, an ultra-short-acting beta1-blocker, is useful for managing supraventricular tachyarrhythmias in sepsis. World journal of critical care medicine 2015; 4(3):251-257

Papathanasiou A, Roustanis E, Zikou X, Galiatsou E, et al: The burden of new onset atrial fibrillation in the intensive care unit (ICU). an observational, prospective, single-center study. Intensive Care Medicine Experimental 2015; 3(Supplement 1):A212

Personett Heather A, Smoot Dustin L, Stollings Joanna L, Sawyer M, et al: Intravenous metoprolol versus diltiazem for rate control in noncardiac, nonthoracic postoperative atrial fibrillation. The Annals of pharmacotherapy 2014; 48(3):314-319

Philip I, Allou N, Mouren S, Bourel P: Atrial fibrillation and anaesthesia-intensive care: An update. Fibrillation auriculaire et anesthesie-reanimation: Mise au point 2010; 22(6):311-320

Pinski SL: Atrial fibrillation in the surgical intensive care unit: Common but understudied. CRITICAL CARE MEDICINE 2004; 32:890-891

Poveda-Jaramillo R, Monaco F, Zangrillo A, Landoni G: Ultra-Short-Acting beta-Blockers (Esmolol and Landiolol) in the Perioperative Period and in Critically Ill Patients. JOURNAL OF CARDIOTHORACIC AND VASCULAR ANESTHESIA 2018; 32:1415-1425

Pravinkumar E: Rate and rhythm are important in critically ill patients. [Comment on: BMJ 2003 Jun 28;326(7404):1411-2; PMID: 12829529 [https://wwwncbinlmnihgov/pubmed/12829529]] 2003; 327(7420):931-932

Pye M, Camm AJ: Use of antiarrhythmic drugs in intensive care. Clinical Intensive Care 1994; 5(6):303-310

Quasim T, Riddell L, Kinsella J: ATRIAL FIBRILLATION IN THE INTENSIVE CARE UNIT. CRITICAL CARE MEDICINE 2010; 38:U124-U124

Quon Michael J, Behlouli H, Pilote L: Anticoagulant Use and Risk of Ischemic Stroke and Bleeding in Patients With Secondary Atrial Fibrillation Associated With Acute Coronary Syndromes, AcutePulmonary Disease, or Sepsis. JACC Clinical electrophysiology 2018; 4:386-393

Ratnaparkhi AB, Walton J: THE PRACTICE OF THE USE OF ANTICOAGULATION FOR ACUTE-ONSET ATRIAL FIBRILLATION IN THE INTENSIVE CARE UNITS OF THE NORTH EAST REGION OF UK; A REGIONAL AUDIT. INTENSIVE CARE MEDICINE 2010; 36:S124-S124

Richards KJC, Cohen AT: Cardiac arrhythmias in the critically ill. Anaesthesia and Intensive Care Medicine 2006; 7(8):289-293

Rudiger A, Breitenstein A, Arrigo M, Salzberg S, et al: Suitability, safety and efficacy of vernakalant for new onset atrial fibrillation in critically ill patients. 26th Annual Congress of the European Society of Intensive Care Medicine, ESICM 2013 Paris France 2013; 39(SUPPL. 2):S291

Salem J-E, El-Aissaoui M, Alazard M, Hulot J-S, et al: Modeling of Amiodarone Effect on Heart Rate Control in Critically Ill Patients with Atrial Tachyarrhythmias. Clinical Pharmacokinetics 2016; 55(8):991-1002

Salem J-E, Dureau P, Funck-Brentano C, Hulot J-S, et al: Effectiveness of heart rate control on hemodynamics in critically ill patients with atrial tachyarrhythmias managed by amiodarone. PHARMACOLOGICAL RESEARCH 2017; 122:118-126

Salerno DM: Supraventricular tachyarrhythmias in the intensive care unit. [Comment in: Postgrad Med 1992 Sep 15;92(4):34; PMID: 1528815 [https://wwwncbinlmnihgov/pubmed/1528815]] 1992; 91(6):293-210

Salman Salam S, Bajwa Abubakr A, Afessa B: Paroxysmal atrial fibrillation in patients with sepsis admitted to the intensive care unit (ICU). CHEST 2006; 130:225S-225S

Samarin Michael J, Mohrien Kerry M, Oliphant Carrie S: Continuous Intravenous Antiarrhythmic Agents in the Intensive Care Unit Strategies for Safe and Effective Use of Amiodarone, Lidocaine, and Procainamide. CRITICAL CARE NURSING QUARTERLY 2015; 38:329-344

Sanai L, Armstrong IR: Supraventricular tachydysrhythmias in the critically ill. A review of antidysrhythmic therapy in patients with SVT. British Journal of Intensive Care 1993; 3(10):358

Sargent DA: Using ibutilide to convert atrial fibrillation and flutter. Dimensions of critical care nursing : DCCN 1999; 18(4):2-12

Schoaps R, Donovan J, Hazard S, Karamchandani K: Anticoagulation on hospital discharge in critically ill patients with new-onset atrial fibrillation. 46th Critical Care Congress of the Society of Critical Care Medicine, SCCM 2016 United States 2016; 44(12 Supplement 1):128

Seemann A, Boissier F, Razazi K, Carteaux G, et al: New-onset supraventricular arrhythmia during septic shock: prevalence, risk factors and prognosis. Annals of Intensive Care 2015; 5:27-27

Seguin P, Laviolle B, Maurice A, Leclercq C, et al: Atrial fibrillation in trauma patients requiring intensive care. Intensive Care Medicine 2006; 32:398-404

Sekhri A, Aronow WS, Sekhri V, Palaniswamy C, et al: Treatment of patients with supraventricular tachyarrhythmias in a medical intensive care unit. Comprehensive Therapy 2009; 35(3-4):188-191

Sekhri A, Aronow WS, Sekhri V, Palaniswamy C, et al: Treatment of supraventricular tachyarrhythmias in a medical intensive care unit supervised by a pulmonary critical care specialist. American College of Chest Physicians Annual Meeting, CHEST 2009 San Diego, CA United States 2009; 136(4)

Sen P, Kundu A, Sardar P, Chetterjee S, et al: Outcomes after cardioversion in patients treated with non vitamin-K antagonist oral anti coagulants (NOACS)-insights from a meta analysis. American Thoracic Society International Conference, ATS 2015 Denver, CO United States 2015; 191(MeetingAbstracts)

Shah A, Pillai MV, Gandhi U, Eden E, et al: New-Onset Atrial Fibrillation/atrial Flutter In Medical Intensive Care Unit (MICU) Patients With Severe Sepsis Is Associated With Stroke And Increased Mortality. AMERICAN JOURNAL OF RESPIRATORY AND CRITICAL CARE MEDICINE 2014; 189

Shaver Ciara M, Chen W, Janz David R, May Addison K, et al: Atrial fibrillation is an independent predictor of mortality in critically ill patients. Critical Care Medicine 2015; 43:2104-2111

Shibata Sho C, Uchiyama A, Ohta N, Fujino Y: Efficacy and Safety of Landiolol Compared to Amiodarone for the Management of Postoperative Atrial Fibrillation in Intensive Care Patients. Journal of cardiothoracic and vascular anesthesia 2016; 30(2):418-422

Smith H, Yeung C, Gowing S, Sadek M, et al: A review and analysis of strategies for prediction, prevention and management of post-operative atrial fibrillation after non-cardiac thoracic surgery. Journal of thoracic disease 2018; 10(Suppl 32):S3799-s3808

Joseph Thomas T, DiMeglio M, Huffenberger A, Laudanski K: Behavioural patterns of electrolyte repletion in intensive care units: lessons from a large electronic dataset. SCIENTIFIC REPORTS 2018; 8

Tober K, Quasim T, Kinsella J: ATRIAL FIBRILLATION; HAEMODYNAMICS AND MORTALITY IN POST-OPERATIVE PATIENTS ON A GENERAL INTENSIVE CARE UNIT. INTENSIVE CARE MEDICINE 2012; 38:S159-S159

Tracy C, Boushahri A: Managing Arrhythmias in the Intensive Care Unit. CRITICAL CARE CLINICS 2014; 30:365+-365+

Trappe H-J: Treating critical supraventricular and ventricular arrhythmias. Journal of emergencies, trauma, and shock 2010; 3(2):143-152

Trappe H-J, Brandts B, Weismueller P: Arrhythmias in the intensive care patient. Current opinion in critical care 2003; 9(5):345-355

Trohman RG: Supraventricular tachycardia: Implications for the intensivist. CRITICAL CARE MEDICINE 2000; 28:N129-N135

Trottier S, Bowie D, Stovall M: Acute atrial fibrillation in the ICU: Therapeutic interventions and hospital course. 42nd Critical Care Congress of the Society of Critical Care Medicine, SCCM 2013 San Juan Puerto Rico 2012; 40(12 SUPPL. 1):67

Van der Does WFB, De Groot NMS: Prophylaxis with amiodarone for postoperative atrial fibrillation: When and who? Journal of Thoracic Disease 2018; 10(Supplement33):S3831-S3833

Varriale P, Sedighi A: Acute management of atrial fibrillation and atrial flutter in the critical care unit: should it be ibutilide? Clinical cardiology 2000; 23(4):265-268

Walkey AJ, Benjamin EJ, Lubitz SA: New-onset atrial fibrillation during hospitalization. Journal of the American College of Cardiology 2014; 64(22):2432-2433

Walkey AJ, Evans SR, Winter M, Benjamin E: Comparative effectiveness of heart rate control medications for atrial fibrillation during sepsis: A propensity-matched cohort study. American Thoracic Society International Conference, ATS 2015 Denver, CO United States 2015; 191(MeetingAbstracts)

Walkey AJ, McManus D: When Rhythm Changes Cause the Blues: New-Onset Atrial Fibrillation during Sepsis. American Journal of Respiratory and Critical Care Medicine 2017; 195(2):152-153

Willich T, Hammwohner M, Goette A: Therapy of atrial fibrillation in the critically ill. Therapie des Vorhofflimmern beim kritisch Kranken 2012; 107(5):368-376

Wong A, Pierce T: Cardiac arrhythmias in the critically ill. Anaesthesia and Intensive Care Medicine 2012; 13(8):360-368

Wood M, Thompson J: Drugs acting on the heart: anti-arrhythmics. Anaesthesia and Intensive Care Medicine 2009; 10(8):388-391

Yokota T, Uchino S, Yoshida T, Fujii T, et al: Predictors for sustained new-onset atrial fibrillation in critically ill patients: a retrospective observational study. JOURNAL OF ANESTHESIA 2018; 32:681-687

Yoshida T, Uchino S, Yokota T, Fujii T, et al: The impact of sustained new-onset atrial fibrillation on mortality and stroke incidence in critically ill patients: A retrospective cohort study. JOURNAL OF CRITICAL CARE 2018; 44:267-272

Yucel E, Hollenberg S: Atrial Fibrillation in Critical Illness: Innocent Bystander or Guilty Party? Critical Care Medicine 2015; 43(10):2254-2255

Zochios VA, Wilkinson J: Correspondence regarding: Atrial fibrillation in the intensive care setting. Journal of the Intensive Care Society 2013; 14(3):276-277

# References

1. Balser JR, Martinez EA, Winters BD, Perdue PW, et al: Beta-adrenergic blockade accelerates conversion of postoperative supraventricular tachyarrhythmias. *Anesthesiology* 1998; 89(5):1052-1059

2. Delle Karth G, Geppert A, Neunteufl T, Priglinger U, et al: Amiodarone versus diltiazem for rate control in critically ill patients with atrial tachyarrhythmias. *Critical care medicine* 2001; 29(6):1149-1153

3. Gerlach A, Kane-Gill S, F Dasta J, Steinberg S, et al: Diltiazem versus Amiodarone for New-Onset Atrial Arrhythmias in Non-Cardiac Post Surgical Patients: A Cohort Study. 2008

4. Launey Y, Lasocki S, Asehnoune K, Gaudriot B, et al: Impact of Low-Dose Hydrocortisone on the Incidence of Atrial Fibrillation in Patients With Septic Shock: A Propensity Score-Inverse Probability of Treatment Weighting Cohort Study. *Journal of intensive care medicine* 2019; 34(3):238-244

5. Balik M, Kolnikova I, Maly M, Waldauf P, et al: Propafenone for supraventricular arrhythmias in septic shock-Comparison to amiodarone and metoprolol. *Journal of critical care* 2017; 41:16-23

6. Cho MS, Nam G-B, Kim YN, Hwang J, et al: Abstract 20382: Comparison of Outcomes After Rhythm or Rate Control Strategy for the New-Onset Atrial Fibrillation in Critically Ill Sepsis Patients Managed in the Medical Intensive Care Unit. *Circulation* 2017; 136(suppl_1):A20382-A20382

7. Jaffer F, Anand S, Ajay-obe A, Parbtani R, et al: Use of Amiodarone in Management of Atrial Tachyarrhythmia in Septic Shock. *CHEST* 2016; 150(4):361A

8. Kane S, Hanes S (Eds): 946: IMPACT OF HYDROCORTISONE ON ATRIAL FIBRILLATION IN SEPTIC SHOCK. 2014

9. Matsumoto Y, Shibata SC, Maeda A, Imada T, et al: Comparison of the short acting beta1 blocker landiolol with amiodarone for pharmacological cardioversion for atrial fibrillation in intensive care unit patients. *2015 Annual Meeting of the International Anesthesia Research Society, IARS 2015 Honolulu, HI United States* 2015; 120(3 SUPPL. 1):S109

10. Brown M, Nassoiy S, Chaney W, Plackett TP, et al: Impact and treatment success of new-onset atrial fibrillation with rapid ventricular rate development in the surgical intensive care unit. *The Journal of surgical research* 2018; 229:66-75

11. Mieure KD, Moranville MP, Park JJ, Lat I, et al: A comparison of intravenous rate control agents for new onset atrial fibrillation with rapid ventricular rate. *32nd Annual Scientific Sessions of the Heart Rhythm Society, Heart Rhythm San Francisco, CA United States* 2011; 8(5 SUPPL. 1):S216-S217

12. Walkey AJ, Evans SR, Winter MR, Benjamin EJ: Practice Patterns and Outcomes of Treatments for Atrial Fibrillation During Sepsis: A Propensity-Matched Cohort Study. *Chest* 2016; 149(1):74-83

13. Walkey AJ, Quinn EK, Winter MR, McManus DD, et al: Practice Patterns and Outcomes Associated With Use of Anticoagulation Among Patients With Atrial Fibrillation During Sepsis. *JAMA Cardiology* 2016; 1(6):682-690

14. Delle Karth G, Schillinger M, Geppert A, Haumer M, et al: Ibutilide for rapid conversion of atrial fibrillation or flutter in a mixed critically ill patient population. *Wiener klinische Wochenschrift* 2005; 117(3):92-97

15. Hennersdorf MG, Perings SM, Zuhlke C, Heidland UE, et al: Conversion of recent-onset atrial fibrillation or flutter with ibutilide after amiodarone has failed. *Intensive care medicine* 2002; 28(7):925-929

16. Mayr A, Ritsch N, Knotzer H, Dunser M, et al: Effectiveness of direct-current cardioversion for treatment of supraventricular tachyarrhythmias, in particular atrial fibrillation, in surgical intensive care patients. *Critical care medicine* 2003; 31(2):401-405

17. Nakamura K, Inokuchi R, Hiruma T, Tokunaga K, et al: Switching therapy from intravenous beta blocker to bisoprolol transdermal patch for atrial fibrillation tachycardia. *Journal of anesthesia* 2016; 30(5):891-894

18. Slavik RS, de Lemos J, Gorman SK, Buchkowsky S: Patterns of practice for the acute management of new-onset atrial fibrillation in critically-ill intensive care unit patients. *Critical care medicine* 2003; 31(12):A137-A137

19. Sleeswijk ME, Tulleken JE, Van Noord T, Meertens JH, et al: Efficacy of magnesium-amiodarone step-up scheme in critically ill patients with new-onset atrial fibrillation: a prospective observational study. *Journal of intensive care medicine* 2008; 23(1):61-66

20. Burris JM, Subramanian A, Sansgiry S, Palacio CH, et al: Perioperative atrial arrhythmias in noncardiothoracic patients: a review of risk factors and treatment strategies in the veteran population. *American journal of surgery* 2010; 200(5):601-605

21. Kanji S, Williamson DR, Yaghchi BM, Albert M, et al: Epidemiology and management of atrial fibrillation in medical and noncardiac surgical adult intensive care unit patients. *Journal of critical care* 2012; 27(3):326.e321-328

22. Kyo M, Hosokawa K, Ohshimo S, Kida Y, et al: High serum potassium level is associated with successful electrical cardioversion for new-onset atrial fibrillation in the intensive care unit: A retrospective observational study. *Anaesthesia and intensive care* 2019; 47(1):52-59

23. Liu WC, Lin WY, Lin CS, Huang HB, et al: Prognostic impact of restored sinus rhythm in patients with sepsis and new-onset atrial fibrillation. *Critical care (London, England)* 2016; 20(1):373

24. Mayr AJ, Dunser MW, Ritsch N, Pajk W, et al: High-dosage continuous amiodarone therapy to treat new-onset supraventricular tachyarrhythmias in surgical intensive care patients: an observational study. *Wiener klinische Wochenschrift* 2004; 116(9-10):310-317

25. Mitric G, Udy A, Bandeshe H, Clement P, et al: Variable use of amiodarone is associated with a greater risk of recurrence of atrial fibrillation in the critically ill. *Critical care* 2016; 20:90

26. Bedford JP, Harford M, Petrinic T, Young JD, et al: Risk factors for new-onset atrial fibrillation on the general adult ICU: A systematic review. *Journal of critical care* 2019; 53:169-175

27. Yoshida T, Fujii T, Uchino S, Takinami M: Epidemiology, prevention, and treatment of new-onset atrial fibrillation in critically ill: a systematic review. *Journal of intensive care* 2015; 3(1):19

28. Balik M, Matousek V, Maly M, Brozek T: Management of arrhythmia in sepsis and septic shock. *Anaesthesiology intensive therapy* 2017; 49(5):419-429

29. Bosch NA, Cimini J, Walkey AJ: Atrial Fibrillation in the ICU. *Chest* 2018; 154(6):1424-1434

30. Kanji S, Stewart R, Fergusson DA, McIntyre L, et al: Treatment of new-onset atrial fibrillation in noncardiac intensive care unit patients: a systematic review of randomized controlled trials. *Critical care medicine* 2008; 36(5):1620-1624

31. Keller M, Meierhenrich R: [New onset atrial fibrillation in patients with sepsis]. *Der Anaesthesist* 2017; 66(10):786-794

32. Rehberg S, Joannidis M, Whitehouse T, Morelli A: Landiolol for managing atrial fibrillation in intensive care. *European heart journal supplements : journal of the European Society of Cardiology* 2018; 20(Suppl A):A15-a18

33. Schwartz A, Brotfain E, Koyfman L, Klein M: Cardiac Arrhythmias in a Septic ICU Population: A Review. *Journal of critical care medicine (Universitatea de Medicina si Farmacie din Targu-Mures)* 2015; 1(4):140-146

34. Shapiro WA, Schulze K: An Algorithm for the Practical Management of the New Onset of Atrial Fibrillation in Patients Admitted to the ICU. *ICU Director* 2012; 3(4):179-184

35. Sibley S, Muscedere J: New-onset atrial fibrillation in critically ill patients. *Can Respir J* 2015; 22(3):179-182

36. Sleeswijk ME, Van Noord T, Tulleken JE, Ligtenberg JJ, et al: Clinical review: treatment of new-onset atrial fibrillation in medical intensive care patients--a clinical framework. *Critical care (London, England)* 2007; 11(6):233

37. Tran B: Atrial Fibrillation in Sepsis: Should We Worry? *Relias Media* 2018

38. Labbe V, Ederhy S, Fartoukh M, Cohen A: Should we administrate anticoagulants to critically ill patients with new onset supraventricular arrhythmias? *Archives of cardiovascular diseases* 2015; 108(4):217-219

39. Chean CS, McAuley D, Gordon A, Welters ID: Current practice in the management of new-onset atrial fibrillation in critically ill patients: a UK-wide survey. *PeerJ* 2017; 5:e3716

40. Walkey AJ, Hogarth DK, Lip GYH: Optimizing atrial fibrillation management: from ICU and beyond. *Chest* 2015; 148(4):859-864

41. Vieillard-Baron A, Boyd J: Non-antiarrhythmic interventions in new onset and paroxysmal sepsis-related atrial fibrillation. *Intensive care medicine* 2018; 44(1):94-97

42. Walton J: The need for adequate rate control points to amiodarone as the drug of choice for treating critically ill patients with acute onset atrial fibrillation. *Critical care medicine* 2008; 36(11):3132-3133

43. Trohman RG: Atrial fibrillation in the critically ill: common sense for a common problem. *Critical care medicine* 2008; 36(5):1681-1682

1. Hydrocortisone [↑](#footnote-ref-2)
2. Includes calcium channel blockers and amiodarone (see T groups [↑](#footnote-ref-3)
3. Relative risk [↑](#footnote-ref-4)
4. Relative risk [↑](#footnote-ref-5)
